# A new lysine biosynthetic enzyme from a bacterial endosymbiont shaped by genetic drift and genome reduction

**DOI:** 10.1002/pro.5083

**Published:** 2024-06-26

**Authors:** Jenna M. Gilkes, Rebekah A. Frampton, Amanda J. Board, André O. Hudson, Thomas G. Price, Vanessa K. Morris, Deborah L. Crittenden, Andrew C. Muscroft‐Taylor, Campbell R. Sheen, Grant R. Smith, Renwick C. J. Dobson

**Affiliations:** ^1^ Biomolecular Interaction Centre School of Biological Sciences, University of Canterbury Christchurch New Zealand; ^2^ The New Zealand Institute for Plant and Food Research Limited Lincoln New Zealand; ^3^ Callaghan Innovation, University of Canterbury Christchurch New Zealand; ^4^ Rochester Institute of Technology, Thomas H. Gosnell School of Life Sciences Rochester New York USA; ^5^ Biomolecular Interaction Centre School of Chemical and Physical Sciences, University of Canterbury Christchurch New Zealand; ^6^ Bio21 Molecular Science and Biotechnology Institute, Department of Biochemistry and Molecular Biology University of Melbourne Parkville Victoria Australia

**Keywords:** *Candidatus* Liberibacter solanacearum, dihydrodipicolinate synthase, enzyme evolution, enzyme mechanism, lysine biosynthesis

## Abstract

The effect of population bottlenecks and genome reduction on enzyme function is poorly understood. *Candidatus* Liberibacter solanacearum is a bacterium with a reduced genome that is transmitted vertically to the egg of an infected psyllid—a population bottleneck that imposes genetic drift and is predicted to affect protein structure and function. Here, we define the function of *Ca*. L. solanacearum dihydrodipicolinate synthase (*C*LsoDHDPS), which catalyzes the committed branchpoint reaction in diaminopimelate and lysine biosynthesis. We demonstrate that *C*LsoDHDPS is expressed in *Ca*. L. solanacearum and expression is increased ~2‐fold in the insect host compared to *in planta*. *C*LsoDHDPS has decreased thermal stability and increased aggregation propensity, implying mutations have destabilized the enzyme but are compensated for through elevated chaperone expression and a stabilized oligomeric state. *C*LsoDHDPS uses a ternary‐complex kinetic mechanism, which is to date unique among DHDPS enzymes, has unusually low catalytic ability, but an unusually high substrate affinity. Structural studies demonstrate that the active site is more open, and the structure of *C*LsoDHDPS with both pyruvate and the substrate analogue succinic‐semialdehyde reveals that the product is both structurally and energetically different and therefore evolution has in this case fashioned a new enzyme. Our study suggests the effects of genome reduction and genetic drift on the function of essential enzymes and provides insights on bacteria‐host co‐evolutionary associations. We propose that bacteria with endosymbiotic lifestyles present a rich vein of interesting enzymes useful for understanding enzyme function and/or informing protein engineering efforts.

## 1 INTRODUCTION

Bacteria evolve to occupy a broad range of niches that include extreme temperatures and pH fluxes. Some cultivate complex interactions with other organisms; for example, the obligate endosymbiotic associations bacteria have formed with plant phloem feeding insects (Nakabachi et al., 2006). Such interactions result in co‐speciation of the bacteria endosymbiont and insect host that led to some bacteria reducing their genome size. This is thought to reflect the richness of host metabolites that allow for the loss of genes previously essential to the bacterial endosymbiont (Douglas, 2006; Ibanez et al., 2014; Marais et al., 2008; Thao et al., 2000). The benefit of the relationship to the host insect is the acquisition of metabolic capabilities from the bacterial endosymbiont that are essential for the insect when feeding on nutrient limited sources, such as the phloem of plants (Wu et al., 2006).

**FIGURE 1:**
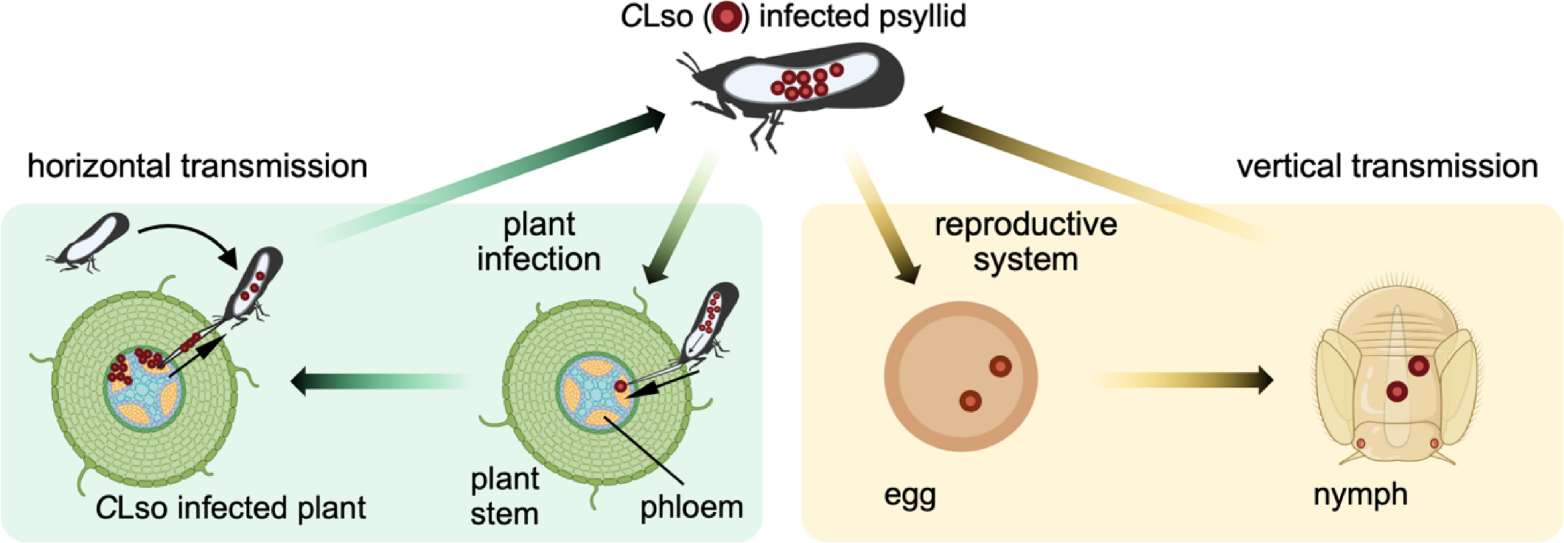
The lifecycle of *Ca*. L. solanacearum and its psyllid vector, *B. cockerelli*. Horizontal transmission, green box. A *Ca*. L. solanacearum infected psyllid feeds on the phloem sap of a health plant. *Ca*. L. solanacearum is transmitted via salivary secretions into a plant during psyllid feeding, which leads to *Ca*. L. solanacearum infection of the plant. A *Ca*. L. solanacearum free psyllid can then become infected upon feeding on the phloem of an infected plant. *Ca*. L. solanacearum within the gut can pass through the alimentary canal wall, move through the hemolymph to reach the salivary glands ready for reinfection. Vertical transmission, orange box. *Ca*. L. solanacearum are transmitted to the offspring (egg and nymph) of an infected female psyllid.

*Candidatus* Liberibacter solanacearum is an unculturable, reduced genome (1.26 Mbp, cf. 5.6 Mbp for *Escherichia coli*), Gram‐negative α‐proteobacterium (Lin et al., 2011). It causes zebra chip disease in plants, a major problem in potato‐growing areas causing significant economic impact on the potato industries in North and Central America, Europe, and New Zealand (Munyaneza, 2010; Munyaneza et al., 2007; Secor et al., 2006, and two useful reviews Munyaneza, 2012; Vereijssen et al., 2018). *Ca*. L. solanacearum persists in two diverse, but nutrient limiting niches: as an insect endosymbiont and as a phytopathogen within the phloem of solanaceous plants. It is transmitted through these niches either horizontally, or vertically (Figure 1).

During horizontal transmission, the psyllid acquires the bacterium by feeding on infected solanaceous plants (Figure 1, green box). The bacterium is then vectored by the insect *Bactericera cockerelli* (Šulc) (Hemiptera: Triozidae), commonly known as the tomato potato psyllid (Buchman et al., 2011, 2012; Munyaneza, 2010, 2012; Munyaneza et al., 2007). To reinfect the plant, *Ca*. L. solanacearum crosses the insect alimentary canal wall and moves through the hemolymph to the salivary glands where the bacteria can then be transmitted to new host plants during psyllid feeding (Cicero et al., 2017; Pitman et al., 2011; Vereijssen et al., 2018).

During vertical transmission, bacteria are transmitted to the offspring of an infected female psyllid and multiply in the nymph and then adult insect (Figure 1, orange box) (Casteel et al., 2012; Hansen et al., 2008). Fluorescence in situ hybridization determined the spatial localization of *Candidatus* Liberibacter asiaticus in the Asian citrus psyllid (Ammar et al., 2011), demonstrating near systemic infection of the psyllid, including the ovaries. In addition, despite being reared on uninfected plant material, 3.6% of combined psyllid offspring (eggs, nymphs, and adults) derived from *Ca*. L. asiaticus infected females tested positive for the bacterium (Pelz‐Stelinski et al., 2010). Taken together, these reports support vertical transmission of *Ca*. L. asiaticus in Asian citrus psyllid populations and it is likely that it also occurs with other Liberibacter species and their insect vectors (Ammar et al., 2011; Kelley & Pelz‐Stelinski, 2019; Pelz‐Stelinski et al., 2010).

Vertical transmission results in a reduction in the population of bacteria transmitted to the insect offspring, a population bottleneck in sustaining symbionts through successive generations (Mira & Moran, 2002; Woolfit & Bromham, 2003). For bacterial endosymbionts with lifestyles such as *C*Lso, there are consequences for their genome and protein complement when compared to free living bacteria. Successive population bottlenecks allow deleterious mutations to become fixed through genetic drift, since natural selection is limited (Andersson & Hughes, 1996; Muller, 1964; Perreau, Patel, et al., 2021; Perreau, Zhang, et al., 2021; Rispe & Moran, 2000; Wernegreen & Moran, 1999) and reviews (McCutcheon & Moran, 2012; Wernegreen, 2002, 2015). Secondly, a consequence of genome reduction is the degradation of genes for DNA repair and a bias towards adenine and thymine nucleotides (Kelkar & Ochman, 2013). This translates to a mutational bias towards hydrophobic residues at the protein level that results in the destabilization of protein structure (Kelkar & Ochman, 2013; O'Fallon, 2008). Furthermore, the fixation of deleterious mutations by genetic drift affects the thermodynamic stability of proteins (van Ham et al., 2003), destabilizes protein secondary structure and makes the proteins prone to misfolding and aggregation (van Ham et al., 2003). For *C*Lso, horizontal transmission (via the plant) likely offsets some of the consequences of vertical transmission. Here, we investigate the effect of population bottlenecks and genome reduction on enzyme function.

**FIGURE 2:**
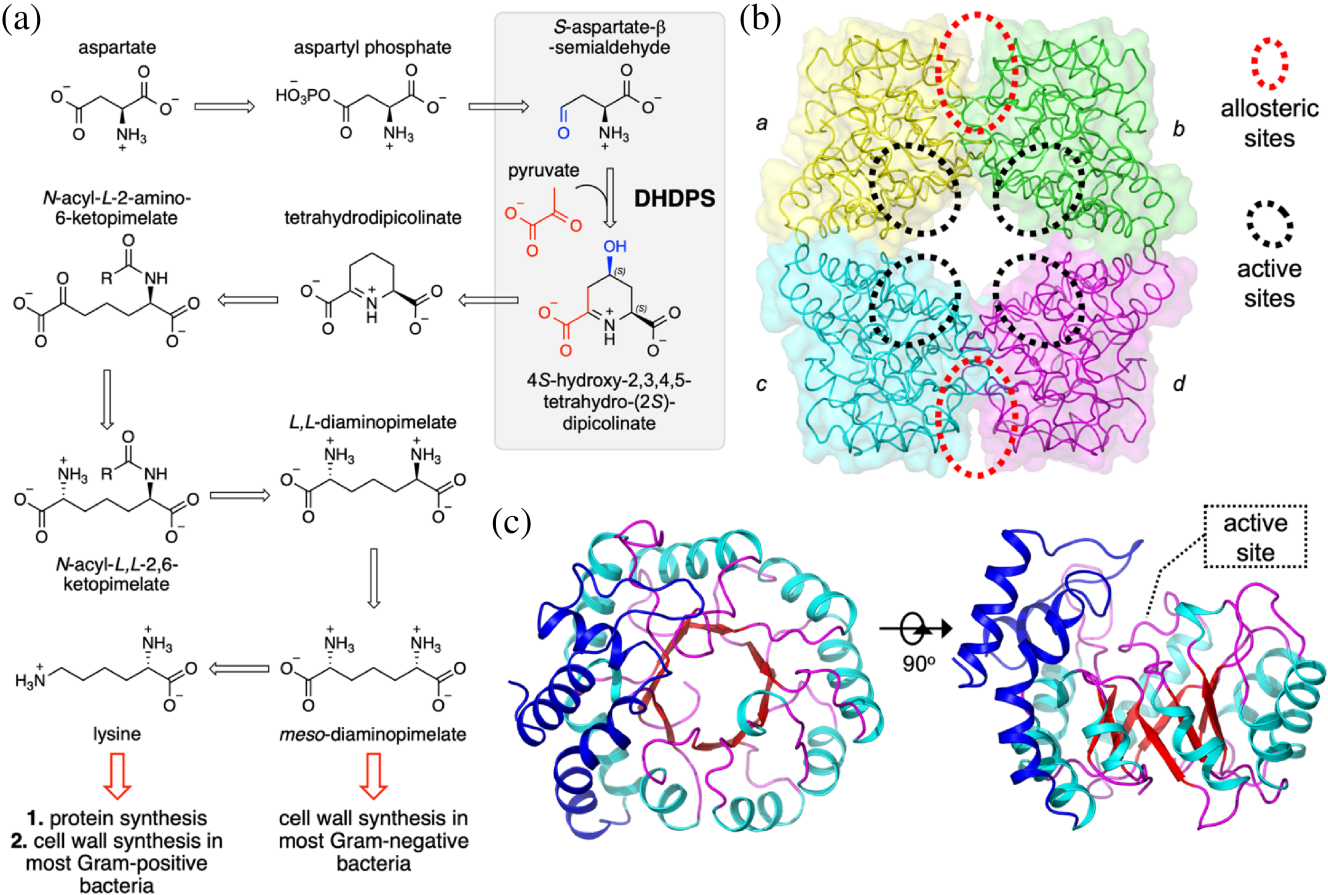
The diaminopimelate pathway and structure of DHDPS. (a) The diaminopimelate pathway that affords precursors for cell wall and protein synthesis. Highlighted in the gray box is the reaction catalyzed by DHDPS—the condensation of pyruvate and *S*‐aspartate‐β‐semialdehyde catalyzed to produce (4*S*)‐hydroxy‐2,3,4,5‐tetrahydro‐(*2S*)‐dipicolinate. (b) The tetrameric structure of the *Ec*DHDPS (PDB ID: 1YXC) with each of the monomers (*a, b, c*, and *d*) differentially colored. The tight‐dimer interface is formed between monomers *a* and *b*, as well as between monomers *c* and *d*. The weak‐dimer interface is formed between monomers *a* and *c*, as well as between *b* and *d*. (c) The TIM barrel structure of the DHDPS monomer, highlighting the location of the active site within the TIM barrel.

*Ca*. L. solanacearum has lost many of the enzymes required for de novo amino acid biosynthesis but is predicted to retain the genes for functional enzymes that synthesize lysine (Lin et al., 2011), suggesting this amino acid pathway is essential. *Ca*. L. solanacearum synthesizes lysine via the diaminopimelate pathway, named after the immediate precursor of lysine, *meso*‐diaminopimelate, which is a major constituent of the bacterial peptidoglycan cell wall in Gram‐negative bacteria [reviewed here Cox, 1996; Cox et al., 2000; Hutton et al., 2007] (Figure 2a). Thus, compounds that inhibit the diaminopimelate pathway could be an effective strategy to control bacterial infections, including *Ca*. L. solanacearum infections in plants, and this has been pursued for several bacterial systems (Boughton et al., 2008; Christoff et al., 2019; Costa et al., 2021; Girodeau et al., 1986; Mitsakos et al., 2008; Turner et al., 2005).

Dihydrodipicolinate synthase (abbreviated to DHDPS) catalyzes the aldol condensation of pyruvate and *S*‐aspartate‐β‐semialdehyde to form (4*S*)‐hydroxy‐2,3,4,5‐tetrahydro‐(2*S*)‐dipicolinate, and this is the first committed step of lysine biosynthesis in the diaminopimelate pathway (Blickling, Renner, et al., 1997; Burgess et al., 2008; Dobson, Valegård, et al., 2004; Domigan et al., 2009; Kefala et al., 2008) (Figure 2a). We note the explicit stereochemistry of the (4*S*)‐hydroxy position, which becomes important later. In plants and Gram‐negative bacteria, DHDPS is allosterically regulated via a negative feedback loop by lysine, the end‐product of the pathway, and therefore a key point of regulation (Atkinson et al., 2012; Dobson et al., 2005; Dobson, Griffin, et al., 2004). The structure of DHDPS has been extensively studied and usually comprises a homotetramer of (β/α)_8_‐barrels that arrange into a dimer of ‘tight‐dimers’ (Figure 2b). The active site is in the center of each (β/α)_8_‐barrel monomer (Figure 2c), while the allosteric lysine‐binding site is situated distal from the active site in the cleft at the tight‐dimer interface, with one lysine binding per monomer, but each lysine molecule being co‐ordinated by residues from each monomer within the tight‐dimer.

The recent development of a method to isolate and characterize recombinant proteins derived from *Ca*. L. solanacearum (Gilkes et al., 2019) provides a unique opportunity to evaluate the evolutionary consequences of highly specialized host‐dependent lifestyles, including understanding the effects of genetic drift and genome evolution on the structure and function of enzymes from reduced genome organisms. Furthermore, because DHDPS has been extensively studied from many bacteria, this enzyme provides an ideal model system for defining the effects of genome reduction and genetic drift on protein structure and enzyme function. Here, we demonstrate that the lifestyle of *Ca*. L. solanacearum has resulted in a DHDPS homologue with highly unusual functional properties, but with surprisingly faithful structural conservation.

## 2 RESULTS

### 2.1 CLsoDHDPS is expressed preferentially within the psyllid, compared to the plant

**FIGURE 3:**
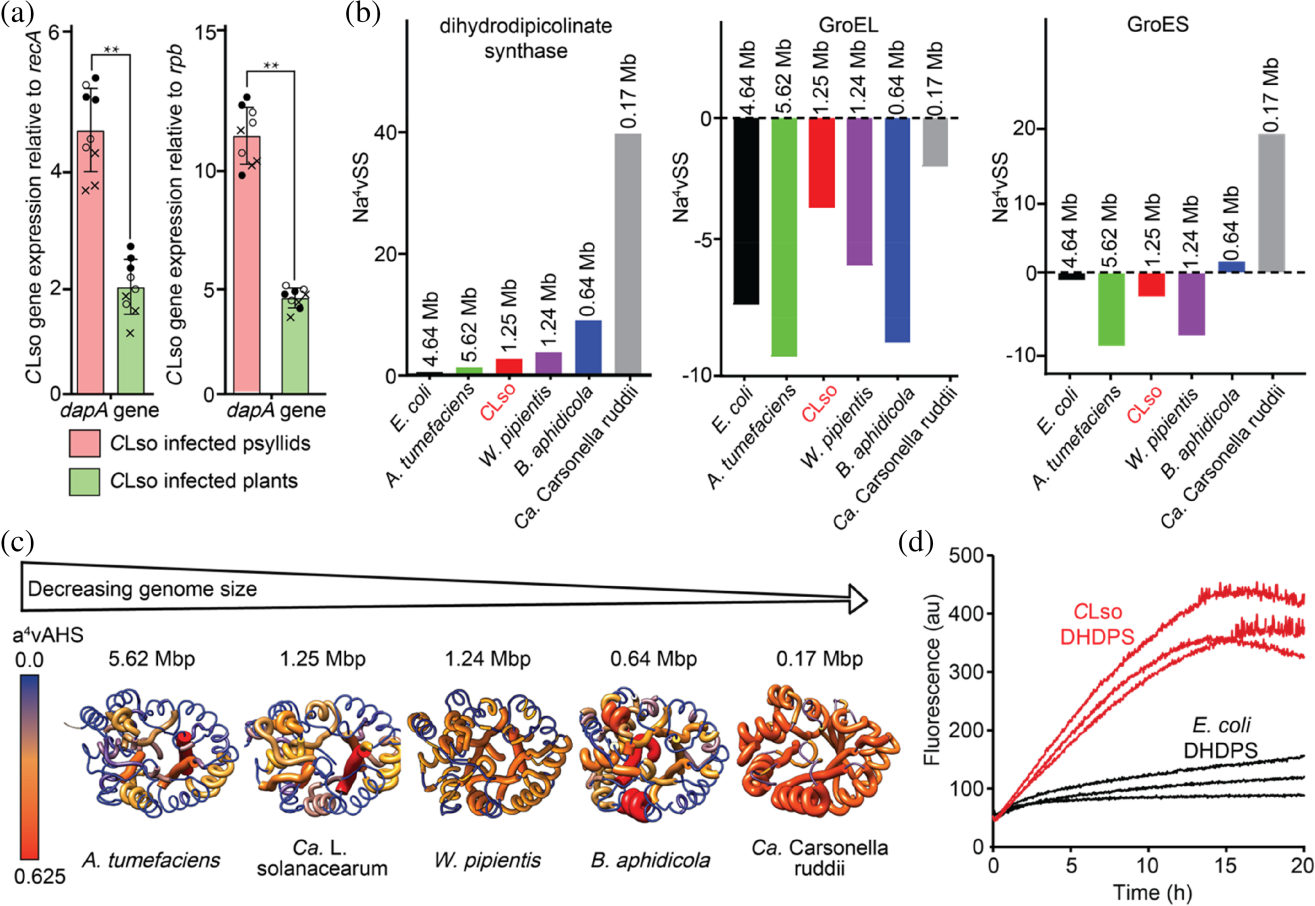
*DapA* gene expression *in psyllids* and *in planta* and aggregation analysis of *Ca. L. solanacearum* proteins. (a) Level of gene expression determined using qRT‐PCR in relation to the housekeeping genes *recA* (recombinase A, KJZ80672, DJ66_1282) and *rpb* (DNA‐directed RNA polymerase beta subunit, KJZ81364.1, DJ66_1258). Mean ± standard deviation for three biological (o, ●, ×) and three technical replicates are plotted. Asterisks indicate statistically significant differences in expression levels between *Ca. L. solanacearum* infected psyllids and plants (**p‐value ≤ 0.01; *t*‐test; *n* = 3). Fold change (log_2_) is the relative gene expression in psyllid versus *in planta* for *C*Lso. Positive values indicate the gene was overexpressed in psyllids compared with *in planta*. The relative gene expression of *dap*A is increased 1.45 ± 1.15 fold (*p*‐value = 0.004) in the psyllid compared to in the plant when normalized to *recA* and 3.2 ± 0.5 fold (*p*‐value = 0.008) when normalized to *rpb*. (b) The aggregation propensity analysis of DHDPS and chaperones GroEL and GroES determined by AGGRESCAN analysis for various organisms. (c) The increase in protein aggregation propensity of the DHDPS monomer with decreasing genome size. The a^4^vAHS is the maximum aggregation propensity value. The structures of the *W. pipientis*, *B. aphidicola*, and *Ca*. Carsonella ruddii DHDPS in the image were generated using SwissModel, whilst the *A. tumefaciens* structure was sourced from the PDB (4I7U) and *C*LsoDHDPS from the structure solved in this study. (d) Thioflavin‐T fluorescence aggregation assay of *E. coli* (black) and *CLso*DHDPS (red) demonstrates increased aggregation propensity for *CLso*DHDPS. Samples contained 33 μM protein with 20 μM thioflavin‐T and were measured in triplicate.

We first verified that the lysine biosynthetic enzyme dihydrodipicolinate synthase (DHDPS) is expressed in *C*Lso. Bacteria adapt to their immediate environment by optimizing their gene expression patterns in response to signals from that environment (Yan et al., 2013): expression of genes necessary in a specific medium is up‐regulated (activated), while those that are unnecessary are down‐regulated (repressed) (Chowdhury et al., 1996). As such, defining gene expression helps to identify those genes necessary for *Ca*. L. solanacearum survival within either the psyllid or plant host. We tested whether the *Ca*. L. solanacearum *dapA* gene, which encodes the enzyme DHDPS, is expressed by the bacterium in native settings (tomato plant and psyllid host) using quantitative reverse transcription‐polymerase chain reaction (qRT‐PCR) to test for the presence of *Ca*. L. solanacearum *dapA* mRNA (Figure 3a). In both environments, we find increased expression levels of *Ca*. L. solanacearum *dapA* mRNA relative to the housekeeping genes *Ca*. L. solanacearum *recA* (recombinase A) and *Ca*. L. solanacearum *rpb* (DNA‐directed RNA polymerase beta subunit), evidence that *dapA* is indeed transcribed. Moreover, the expression of *Ca*. L. solanacearum *dapA* is increased ~2‐fold in the insect host compared to *in planta*, again relative to the housekeeping genes, *recA* and *rpb*. Thus, *dapA* is expressed in *Ca*. L. solanacearum and the level of expression differs when *Ca*. L. solanacearum is in the psyllid gut compared to the phloem of the plant. Without antibodies specific for *C*LsoDHDPS and because *Ca*. L. solanacearum is unculturable and cannot be isolated from the psyllid or plant, it is not possible to verify the protein concentrations of enzyme activity of *C*LsoDHDPS when *Ca*. L. solanacearum is in the psyllid or plant. Currently, the physiological reason/s for the differential expression in the two environments is unknown. We speculate that upregulation of lysine biosynthesis when in the insect gut may reflect increased growth rate and therefore higher needs of *meso‐*diaminopimelate (the cross‐linking amino acid at the third position for the bacterial peptidoglycan cell wall) and lysine (for the synthesis of proteins).

### 2.2 CLsoDHDPS is destabilized and shows increased aggregation propensity

Some bacterial endosymbionts experience population bottlenecks (Mira & Moran, 2002; Woolfit & Bromham, 2003; Workneh et al., 2016) and undergo reductive genome evolution [reviewed in McCutcheon & Moran, 2012; Moran, 2002], which together cause enhanced genomic evolution due to an increased mutation rate (Itoh et al., 2002; Kelkar & Ochman, 2013; Rispe & Moran, 2000). Accumulation of deleterious mutations alter genes and gradually lead to gene loss and mutational meltdown (Andersson & Hughes, 1996; Muller, 1964). These effects are predicted to have a destabilizing effect on protein structure and increase the propensity for aggregation, which in turn may affect protein function (van Ham et al., 2003).

To determine whether the endosymbiont lifestyle has had a destabilizing effect on *C*LsoDHDPS we first used thermal melt analysis, with and without ligands, and compared the data with *E. coli* DHDPS (*Ec*DHDPS, Table 1, Figure Si). Although *C*LsoDHDPS (*T*_m_^D^ = 61.5 ± 0.2°C) has a decreased melting temperature compared to *Ec*DHDPS (*T*_m_^D^ = 64.1 ± 0.2°C), the addition of ligands has a highly destabilizing effect on *C*LsoDHDPS, unlike *Ec*DHDPS. Whereas the addition of pyruvate destabilizes *C*LsoDHDPS by −4.8°C, it stabilizes *Ec*DHDPS by 1.4°C. Lysine has a greater destabilizing effect on *C*LsoDHDPS, decreasing the thermal stability by −7.4°C, but a negligible effect on *Ec*DHDPS. This means that in the presence of ligands, *C*LsoDHDPS is substantially less stable compared to *Ec*DHDPS (−7.5 to −9.3°C). Ligands that thermally destabilize proteins have been reported and a thermodynamic explanation for this behavior put forward (Cimmperman et al., 2008; Kabir et al., 2016; Petrauskas et al., 2024). *C*LsoDHDPS likely samples an ensemble of conformations at room temperature, each of which have different thermal stability. Ligand binding could select a subset of conformations that are susceptible to thermal denaturation compared to the apo ensemble (i.e., decreased stability, *T*_m_^D^). Given that the substrates (especially pyruvate as a metabolite of central metabolism) and lysine are likely to be free in the cell to bind *C*LsoDHDPS, these data demonstrate that *C*LsoDHDPS is significantly destabilized, which hints at a dynamic or functional difference. A survey of the thermal stability for DHDPS homologues in the presence of pyruvate (Table Si) suggests that *C*LsoDHDPS is the least stable homologue studied so far; a caveat for this comparison is that these were determined using different methods and in different conditions (buffers, pH, and protein concentrations). We also assessed the thermal stability on the only other *Ca*. L. solanacearum derived protein to be recombinantly isolated, pyruvate kinase (Gilkes et al., 2019), compared to its *E. coli* homologue (Table Svii). As for *C*LsoDHDPS, we find that *Ca*. L. solanacearum pyruvate kinase is significantly less stable compared to the *E. coli* homologue (−10.1 to −14.4°C), although the significant destabilizing effect by ligands was not evident in this case.

**TABLE 1:** Comparison of first derivative (*T*_m_^D^) melting temperatures for *C*LsoDHDPS and *Ec*DHDPS.

	*C*LsoDHDPS (*T*_m_^D^,°C)		*C*LsoDHDPS–*Ec*DHDPS	*Ec*DHDPS (*T*_m_^D^,°C)		
Apo	61.5 ± 0.2		−2.6	64.1 ± 0.1		
+ substrate		Δ*T*_m_^D^ apo			Δ*T*_m_^D^ apo	
Pyruvate	56.7 ± 0.2	−4.8	−8.8	65.5 ± 0	1.4	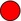 10.0–7.0°C
Lysine	54.1 ± 0.4	−7.4	−9.3	63.4 ± 0.2	−0.7	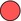 7.0–4.0°C
SSA	55.1 ± 0.2	−6.4	−7.9	63.0 ± 0.1	−1.1	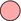 4.0–1.0°C
pyr/SSA	57.7 ± 0.3	−3.8	−8.6	66.3 ± 0.1	2.2	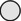 1.0 to −1.0°C
lys/pyr	56.3 ± 0.2	−5.2	−7.6	63.9 ± 0.1	−0.2	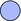 −1.0 to −4.0°C
lys/SSA	54.6 ± 0.4	−6.9	−7.5	62.1 ± 0.1	−2.0	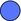 −4.0 to −7.0°C
pyr/lys/SSA	55.9 ± 0.1	−5.6	−9.3	65.2 ± 0	1.1	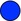 −7.0 to −10.0°C

*Note*: The raw data is shown in Figure Si. Δ*T*_m_^D^ apo refers to the difference in *T*_m_^D^ when combinations of substrates and allosteric effector are added and compared to the apo *T*_m_^D^. Ligands significantly destabilize *C*LsoDHDPS (−4.4 to −7.4°C) but have minimal effect on *Ec*DHDPS (−2.0 to 2.2°C). The ligand concentrations were 10 mM. Errors represent standard errors.

To determine whether the *Ca*. L. solanacearum proteins have a greater propensity for aggregation, AGGREGSCAN (Conchillo‐Solé et al., 2007) was used to predict aggregation‐prone regions in the protein sequences of interest. AGGRESCAN is based on an aggregation‐propensity scale for natural amino acids and assumes that short and specific sequence stretches modulate protein aggregation (Conchillo‐Solé et al., 2007). The intrinsic aggregation properties for DHDPS homologues and the chaperone proteins, GroEL and GroES (discussed in the next section) were predicted and compared across *E. coli* (PDB ID: 1YXC) and *Agrobacterium tumefaciens* (PDB ID: 4I7U), as well as the reduced genome organisms of *Ca*. L. solanacearum (KJZ81861), *Wolbachia pipientis* (KLT22486.1), *Buchnera aphidicola* (BAB12815.1), and *Ca*. Carsonella ruddii (AGS06617.1).

The average aggregation propensity of the sequence (Na^4^vSS) for *C*LsoDHDPS is shown in Figure 3b. DHDPS from the reduced genome organisms *B. aphidicola*, *Ca*. Carsonella ruddii, and *Ca*. L. solanacearum have a greater propensity for protein aggregation when compared against *E. coli* and *A. tumefaciens* (Figure 3b)—here, an increased positive Na^4^vSS score means an increase in the aggregation propensity for the protein. When we broaden the analysis to enzymes within the lysine biosynthetic pathway, we see the same trend (Figure Sii). The aggregation propensity for *C*LsoDHDPS is also demonstrated in Figure 3c, which maps average hot spot area (a^4^vAHS) onto the tertiary structure of the DHDPS enzymes and shows there is an increase in a^4^vAHS as genome size decreases. The *B. aphidicola* and *Ca*. Carsonella ruddii DHDPS enzymes are shown to be most prone to aggregation, in line with the computational studies (van Ham et al., 2003) that suggest that *B. aphidicola* proteins have a reduced and slower protein folding efficiency, increased misfolding and aggregation propensity, or are unstable in their native conformation. Overall, the results are consistent with a decrease in protein stability as genome size decreases.

These findings were experimentally confirmed using Thioflavin‐T aggregation assays comparing *E. coli* and *C*LsoDHDPS (Figure 3d). Although some degree of aggregation could be observed with both forms of DHDPS, the aggregation rate and extent of aggregation were much higher for C*Lso*DHDPS, consistent with a lower protein stability. The half‐time of aggregation of *C*LsoDHDPS was 5.8 ± 0.8 h, while the half time of *Ec*DHDPS aggregation could not be accurately fitted due to lack of clear final plateau within the experimental time frame (20 h).

We next examined and compared the *C*LsoDHDPS protein sequence with that of other bacterial and plant homologues (Figure Siii). In general, there are no obvious sequence differences when comparing *C*LsoDHDPS and homologues from other genome reduced species with well‐established bacterial DHDPS systems. For example, the *C*LsoDHDPS is similar in length (i.e., no deletions or insertions) and generally similar across the sequence (i.e., no areas of obvious difference that map to regions with increased aggregation).

### 2.3 CLsoDHDPS chaperone proteins GroEL and GroES show a low propensity for aggregation

The C*LsoDHDPS* genome has become optimized for growth in the psyllid and plant, yet the effects of genetic drift and genome reduction on bacterial endosymbionts protein complement should lead to an eventual decline in biological fitness. This suggests there is a compensating mechanism to buffer protein destabilization. The retention and increased abundance of chaperone proteins in endosymbionts is thought to assist protein folding and rescue non‐functional protein conformations (Fares et al., 2004; Fares, Barrio, et al., 2002; Fares, Ruiz‐González, et al., 2002; Tokuriki & Tawfik, 2009a). Importantly, chaperones are abundantly expressed and evolutionary conserved among intracellular bacteria (Fares et al., 2004; Fares, Ruiz‐González, et al., 2002; Nachappa et al., 2012; Tokuriki & Tawfik, 2009a).

We previously demonstrated that overexpression and purification of folded and functional recombinant *Ca*. L. solanacearum enzymes, including *C*LsoDHDPS, required the concurrent overexpression of *E. coli* chaperone proteins GroEL and GroES (Gilkes et al., 2019). Moreover, others have shown that the expression of functional *Ec*DHDPS is dependent on the GroEL/GroES chaperone system—disrupting the GroEL/GroES system results in *E. coli* lysis because DHDPS cannot fold and therefore the cell wall constituent diaminopimelate cannot be synthesized (McLennan & Masters, 1998). In the *E. coli* setting, GroEL/ES accelerates the folding of DHDPS 30‐fold by catalyzing segmental structure formation in the TIM‐barrel and lowering the entropy of the energy barrier (Georgescauld et al., 2014). Thus, the requirement to co‐express *E. coli* chaperone proteins to high levels with the *C*LsoDHDPS could indicate the instability of the protein when compared to *Ec*DHDPS, which only requires chromosomal chaperonin expression levels when overexpressed.

It is thought that the abundant overexpression of chaperones in *Ca*. L. solanacearum (Nachappa et al., 2012) assists the folding of conformationally destabilized proteins that occurs as a result of genome reduction and genetic drift (Fares et al., 2004, 2005). Included in Figure 3b is the aggregation propensity for *Ca*. L. solanacearum GroEL and GroES, which suggests that these proteins have decreased aggregation propensity in contrast to the lysine biosynthetic enzymes of *Ca*. L. solanacearum. That is, they retain stability and function. We hypothesize that these *Ca*. L. solanacearum chaperonins buffer against mutational effects and are subject to selection rather than genetic drift, as they are required for stabilizing and folding of *Ca*. L. solanacearum proteins (Fares, Barrio, et al., 2002; Fares, Ruiz‐González, et al., 2002; Moran, 1996; Rutherford, 2003). The GroEL chaperone from all organisms tested showed a low propensity for aggregation. This is consistent with the proposal that *Ca*. L. solanacearum chaperone proteins buffer against the deleterious mutations that accumulate under strong genetic drift from the bacterium's intracellular lifecycle and subsequent fitness decline (Fares, Barrio, et al., 2002; Fares, Ruiz‐González, et al., 2002; Moran, 1996; Rutherford, 2003). Interestingly, biological systems with high GroEL/GroES overexpression increased the number of and buffered against the accumulation of mutations that are destabilizing and increased enzymatic variability (Tokuriki & Tawfik, 2009a, 2009b).

Overall, we demonstrate that *C*LsoDHDPS is destabilized and likely to be more prone to aggregation compared to *E. coli* and *A. tumefaciens*, and more similar in aggregation propensity to other reduced genome bacteria. We also suggest that the GroEL/ES chaperone systems are stable and functional, consistent with the proposal that this is the mechanism by which genome reduced bacteria overcome deleterious mutations. As such, not only is DHDPS expressed in *Ca*. L. solanacearum, but is likely to be in a folded and functional form, facilitated by the GroEL/ES chaperone system.

### 2.4 CLsoDHDPS uses the ternary‐complex kinetic mechanism, but retains allosteric inhibition by lysine

We tested whether genome reduction in *Ca*. L. solanacearum had driven the evolution of altered catalytic functions in *C*LsoDHDPS. Such differences in function could be exploited for designing effective antibacterial agents that target DHDPS, especially if they block an active site that is significantly different to endogenous plant or other bacterial DHDPS enzymes. Recombinant *C*LsoDHDPS was expressed and purified using the method previously described (Gilkes et al., 2019). Initial rate kinetic data were collected using an established assay (Dobson, Valegård, et al., 2004), varying the concentration of both substrates, pyruvate and *S*‐aspartate‐β‐semialdehyde. As a first step, we determined the kinetic mechanism by which *C*LsoDHDPS operates.

**FIGURE 4:**
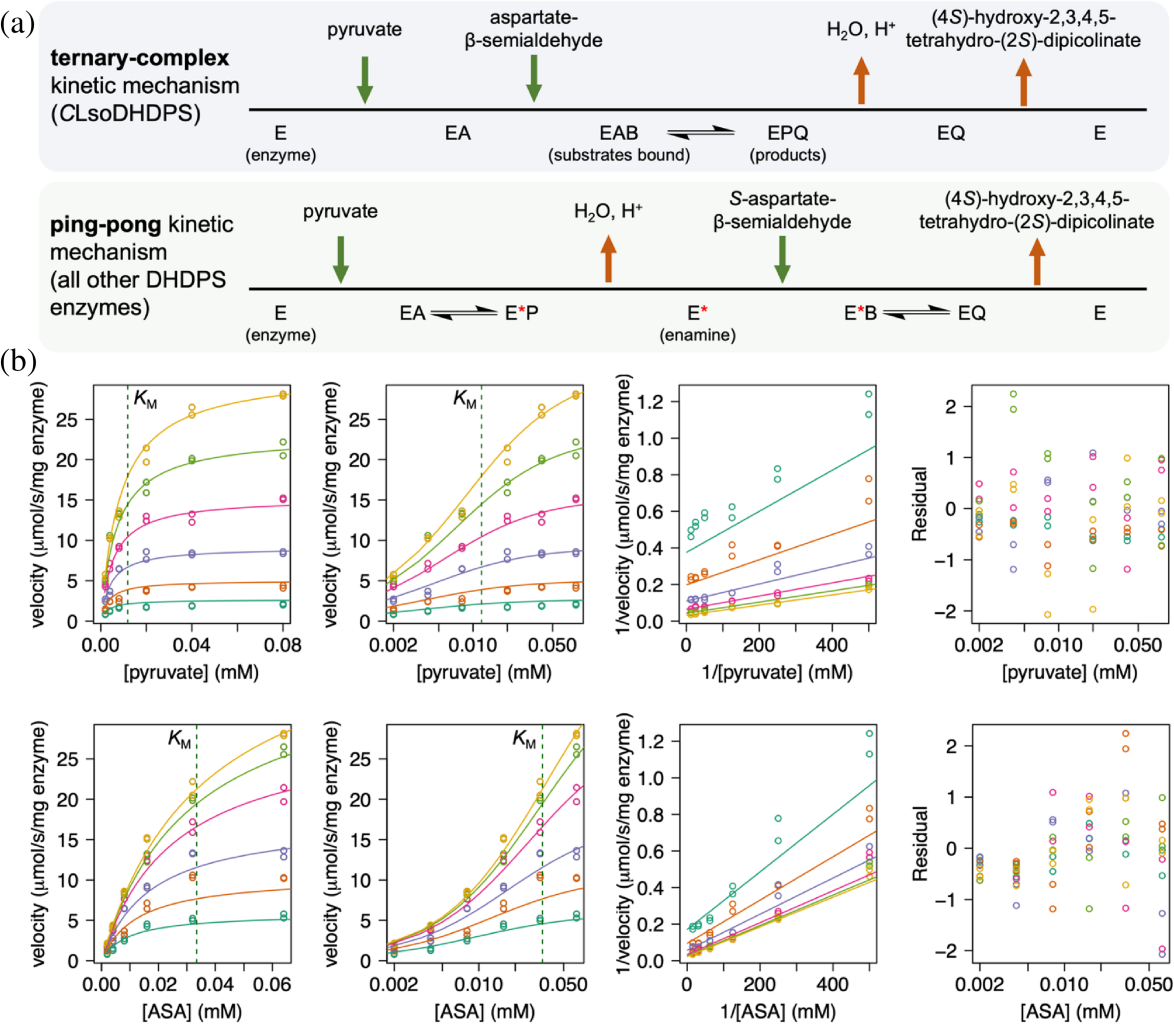
Enzyme kinetic analysis of *C*LsoDHDPS. (a) Schemes for the ternary‐complex and the ping‐pong kinetic models. They differ in the order of substrate binding and product release. (b) Initial rate data best fit to the ternary‐complex kinetic model. The data has an *R* ^2^ value of fit is 0.99. The plots show initial rates with respect to pyruvate (top) and *S*‐aspartate‐β‐semialdehyde (bottom), which were generated using *R* following non‐linear regression of the data against the ternary‐complex model. The four plots from left to right are the direct plot, the direct plot with the substrate concentration on the log scale, the Lineweaver Burk plot, and the residuals of the fit. For the plots with respect to pyruvate (top), the *S*‐aspartate‐β‐semialdehyde concentrations are 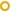 = 0.064 mM, 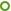 = 0.032 mM, 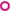 = 0.016 mM, 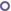 = 0.008 mM, 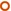
= 0.004 mM, and 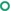 = 0.002 mM. For the plots with respect to *S*‐aspartate‐β‐semialdehyde (*S*‐ASA) (bottom), the pyruvate concentrations are 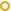 = 0.08 mM, 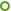 = 0.04 mM, 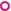 = 0.02 mM, 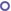 = 0.008 mM,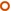 = 0.004 mM, and 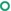 = 0.002 mM. The green dotted line shows the fitted *K*_M_ values for pyruvate (top) and *S*‐aspartate‐β‐semialdehyde (bottom).

Surprisingly, *C*LsoDHDPS follows the ternary‐complex kinetic mechanism (Figure 4a, top), whereas all DHDPS enzymes characterized to date follow the ping‐pong kinetic mechanism (bottom), which suggests that the order of substrate binding and product release has changed and therefore catalytic mechanism is different in this homologue. Initial rate data for varied pyruvate and *S*‐aspartate‐β‐semialdehyde concentrations fit the ternary‐complex model with an *R* ^2^ value of 0.99 (Figure 4b and Table 2). The Akaike test was used to determine the best fit between the ping‐pong and ternary‐complex models and estimated that the probability of the ping‐pong model being correct was <0.01%, whereas the probability of the ternary‐complex model was 99.99% with a difference in the AIC value of 18.9. Unlike the ping‐pong kinetic model, the ternary‐complex kinetic model can be either ordered or random with respect to substrate binding and product release. However, if the Schiff base/enamine needs to form first to enable C—C bond formation in the *C*LsoDHDPS catalytic mechanism, we propose that pyruvate is the first substrate to bind and that H_2_O and H^+^ are the first products released.

**TABLE 2:** Kinetic parameters for *C*LsoDHDPS with respect to pyruvate and *S*‐aspartate‐β‐semialdehyde (*S*‐ASA).

Ternary‐complex model	Fitted kinetic parameters
*k*_cat_ (s^−1^)	6.0 ± 0.2 (5.5–6.5)
*K*_M_^pyruvate^ (mM)	0.012 ± 0.001 (0.009–0.014)
*K*_M_^*S*‐ASA^ (mM)	0.033 ± 0.003 (0.029–0.039)
*k*_cat_/*K*_M_^pyruvate^ (M^−1^ s^−1^)	5.0 × 10^5^
*k*_cat_/*K*_M_^*S*‐ASA^ (M^−1^ s^−1^)	1.8 × 10^5^
*K*_sA_ (mM)	0.0020 ± 0.0007 (0.0013–0.0039)

*Note*: The data were fit to ternary‐complex model using *R* (*R* ^2^ = 0.99), shown in Figure 4. The standard error from the fit for each parameter is shown and the 95% confidence intervals are in brackets.

The kinetic parameters for *C*LsoDHDPS are also surprising. Firstly, the Michaelis constants (*K*_M_) for the substrates were 0.012 ± 0.001 mM with respect to pyruvate and 0.033 ± 0.003 mM with respect to *S*‐aspartate‐β‐semialdehyde—these are considerably lower when compared to the Michaelis constants of other bacterial homologues (Table Sii) and suggest the enzyme has a high affinity for its substrates. Secondly, the turnover number (*k*_cat_) is 6.0 ± 0.2 s^−1^ and is also considerably lower when compared to other bacterial homologues—the only comparable homologue is from *Thermotoga maritima*, but in that case the rates were determined far below the optimal temperature for this enzyme. Since *C*LsoDHDPS uses a ternary‐complex kinetic mechanism, we also report a true substrate dissociation constant for pyruvate (*K*_sA_) of 0.0020 ± 0.0007 mM. Despite the low turnover number, the specificity constants are comparable with other bacterial homologues (Table Sii), driven by improved affinity for the substrates (lower *K*_M_ values). One interpretation is that the accumulation of mutations in *C*LsoDHDPS during genome reduction may have led to a trade‐off between the declining ability to catalyze the chemical transformation, but improved affinity for the substrates in this case. Our kinetic data provides correlative evidence and does not validate this interpretation. However, structural data described in subsequent sections suggest two chemical explanations for this trade‐off.

Next, we determined whether *C*LsoDHDPS retains feedback inhibition by the pathway end‐product, lysine, as seen in other DHDPS homologues studied to date (Dobson, Griffin, et al., 2004; Domigan et al., 2009; Rice et al., 2008). To test this, initial rate data were collected as above, but with varying concentrations of lysine. The initial rate for the enzyme decreases when titrating increasing concentrations of lysine into the assay, demonstrating that *C*LsoDHDPS retains its ability to be allosterically inhibited by lysine (Figure Siv). In addition, lysine acts as a partial inhibitor, since at saturating lysine concentrations some activity is retained, a behavior that is also seen in *Ec*DHDPS (Dobson, Griffin, et al., 2004). Inhibition of *C*LsoDHDPS by lysine when pyruvate is the varied substrate displayed mixed model inhibition with a *K*_ic_ of 0.37 ± 0.07 mM and *K*_iu_ of 0.121 ± 0.005 mM (Table 3). Inhibition of *C*LsoDHDPS by lysine when *S*‐aspartate‐β‐semialdehyde is the varied substrate displayed partial uncompetitive inhibition with a *K*_iu_ of 0.27 ± 0.02 mM (Table 3).

**TABLE 3:** Kinetic parameters for lysine inhibition of *C*LsoDHDPS with respect to pyruvate and *S*‐aspartate‐β‐semialdehyde as a substrate.

	Fitted kinetic parameters	
Varied substrate	Pyruvate	*S*‐Aspartate‐β‐semialdehyde
Kinetic model	Mixed inhibition	Partial uncompetitive inhibition
*K*_M_ (mM)	0.0110 ± 0.0005 (0.010–0.012)	0.022 ± 0.001 (0.020–0.024)
*K*_ic_ (mM)	0.37 ± 0.07 (0.26–0.59)	‐
*K*_iu_ (mM)	0.121 ± 0.005 (0.112–0.131)	0.27 ± 0.02 (0.23–0.31)
*V*_max_ (μmol/min/mg)	32.4 ± 0.5 (31.4–33.4)	33.1 ± 0.8 (31.5–34.7)
*V*_max2_ (μmol/min/mg)	‐	1.0 ± 0.3 (0.5–1.5)
*R* ^2^ for the fit	0.99	0.98

*Note*: The data were fit to respective kinetic models using *R*, as in Figure Siv. The standard error of the fit for each parameter is shown and the 95% confidence intervals are in brackets.

Considering our kinetic experiments together and despite predicting an increased propensity for aggregation, we verify that *C*LsoDHDPS is active, suggesting that although *Ca*. L. solanacearum has lost many of its biosynthetic genes, it has retained lysine biosynthesis. We demonstrate that *C*LsoDHDPS uses a different kinetic mechanism to other DHDPS enzymes, which implies that the catalytic mechanism may be altered in some way, including the order of substrate binding and product release. We also find that the *K*_M_ values for the substrates are considerably lower compared to other homologues, reflecting a higher affinity for the substrates. Although kinetically unusual, *C*LsoDHDPS retains allosteric inhibition by lysine.

### 2.5 Crystal structures of CLsoDHDPS demonstrate a conserved structure, but larger tetrameric interfaces

Next, we solved a series of ligand‐bound *C*LsoDHDPS structures using crystallography to test whether evolutionary pressures resulted in structural changes that account for the altered kinetic behavior. We determined and compared the following X‐ray crystal structures: (1) with the allosteric inhibitor lysine, PDB ID: 7LVL; (2) with the substrate pyruvate, PDB ID: 7LOY; and (3) with pyruvate and succinic semi‐aldehyde, an analogue of the substrate *S*‐aspartate‐β‐semialdehyde, PDB ID: 8GEK. We had difficulty crystallizing apo‐*C*LsoDHDPS, perhaps reflecting some unknown structural heterogeneity that prevented crystal formation. Nonetheless, each of these structures were determined to atomic resolution (1.93–2.40 Å), with sensible geometry and *R*_free_ values (0.209–0.235) (Table Siv). Below, we outline the broad similarities and differences with homologous DHDPS enzymes, before focusing on the active and allosteric sites of the ligand bound structures in later sections.

**FIGURE 5:**
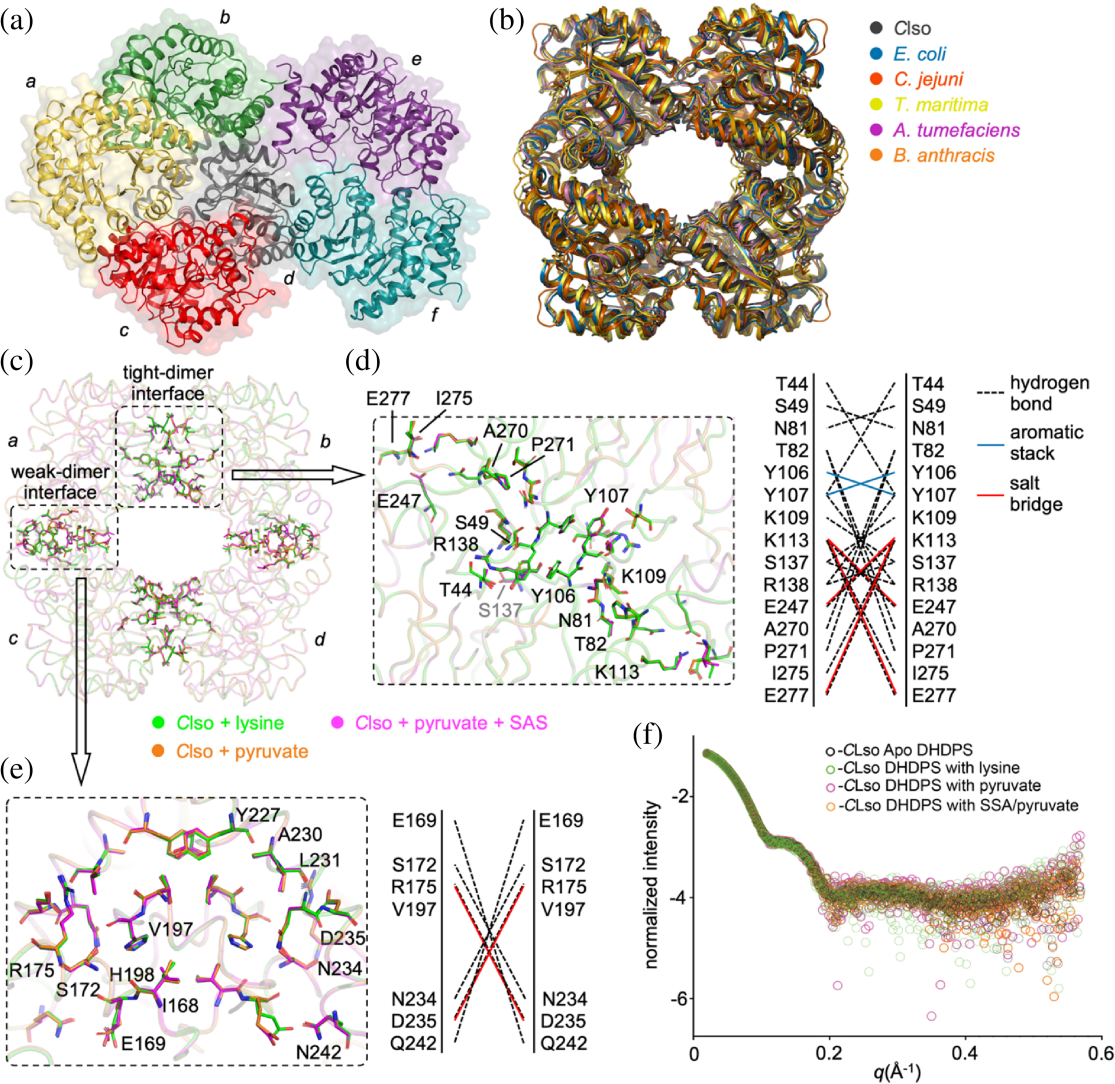
Structure of the *C*LsoDHDPS. (a) The position of the six monomers in the *C*LsoDHDPS asymmetric unit. (b) The *C*LsoDHDPS tetramer (black, PDB ID: 7LOY) aligned with the DHDPS tetramers for *E. coli* (blue, PDB ID: 1YXC, r.m.s.d. = 1.2), *C. jejuni* (red, PDB ID: 4R53, r.m.s.d. = 1.8), *T. maritima* (yellow, PDB ID: 3PB2, r.m.s.d. = 2.2), *A. tumefaciens* (magenta, PDB ID: 4I7U, r.m.s.d. = 1.0), and *B. anthracis* (orange, PDB ID: 3HIJ, r.m.s.d. = 1.1). Monomers are indicated by *a*/*b*/*c*/*d*, as in Figure 2b. (c) An overlay of the liganded *C*LsoDHDPS tetramers. The *C*LsoDHDPS + lysine structure (green) is overlaid with the *C*LsoDHDPS + pyruvate (yellow, r.m.s.d. = 0.14) and the *C*LsoDHDPS + SSA + pyruvate (magenta, r.m.s.d. = 0.16) structures. The structures suggest that ligand binding does not alter the structure of the active and allosteric sites as there is no overall conformational change in the structure (which is confirmed in solution using small angle X‐ray scattering in (f)). The position of the residues at the tight‐dimer interface between monomers *a* and *b* (*c* and *d*) and the residues at the weak dimer interface between monomers *a* and *c* (*b* and *d*) are shown. (d) and (e) focus of the tight dimer and weak dimer interfaces, respectively, as determined by PISA. Broken lines indicate hydrogen‐bonding, red lines indicate salt bridges, and blue lines aromatic stacking. (f) Small angle scattering data comparing the apo *C*LsoDHDPS with ligand bound *C*LsoDHDPS demonstrates that ligand binding does not alter the structure of the enzyme in solution (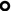 = apo *C*LsoDHDPS, 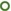 = *C*LsoDHDPS + lysine, 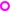 = *C*LsoDHDPS + pyruvate, and 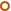 *C*LsoDHDPS + SSA + pyruvate).

We first examined the oligomeric structure of *C*LsoDHDPS, since DHDPS enzymes can form tetrameric (Atkinson et al., 2018; Dobson et al., 2005; Domigan et al., 2009) and/or dimeric structures (Atkinson et al., 2018; Burgess et al., 2008). The oligomeric state is known to be important for function because both the active site and the allosteric binding site comprise residues from the two monomers that form the tight‐interface (the yellow and green monomers in Figure 2b) (Griffin et al., 2008; Pearce et al., 2011). The asymmetric unit for each *C*LsoDHDPS crystal structure contained six monomers that form the typical bacterial tetrameric structure (symmetry operations are needed to complete the second tetramer) (Figure 5a). The tetrameric structure agrees with sedimentation velocity experiments that demonstrate that unliganded *C*LsoDHDPS is tetrameric in solution (Gilkes et al., 2019). A structural alignment of *C*LsoDHDPS with other tetrameric bacterial DHDPS homologues from *E. coli*, *T. maritima*, *Campylobacter jejuni*, *A. tumefaciens*, and *Bacillus anthracis* demonstrates that the *A. tumefaciens* DHDPS tetramer has the closest overall structural similarity, with the lowest root mean square deviation of 1.0 Å (α‐carbons, Figure 5b).

To determine whether binding either the substrates (pyruvate and succinic semi‐aldehyde) or the allosteric inhibitor (lysine) result in subtle conformational changes across the *C*LsoDHDPS tetramer, the ligand bound tetrameric structures were overlayed and changes at the interfaces were investigated (Figure 5c). We note that in all the *C*LsoDHDPS structures presented, the nature of both the tight‐dimer and weak‐dimer interfaces do not change, suggesting that ligand binding does not change the orientation of the monomers within the tetramer, as compared for example with O_2_ binding to hemoglobin.

The tight‐dimer interface between the *a*/*b* (and *c*/*d*) monomers includes residues Tyr106 and Tyr107 that are required for the interlocking of the monomers and contribute to the active site in the opposing monomer (Figure 5d). The Protein Interfaces, Surfaces and Assemblies (PISA) program (Krissinel & Henrick, 2007) was used to calculate the buried surface area at the tight‐dimer interface of *C*LsoDHDPS, which is on average ~1520 Å^2^ (BSA in Table 4) with ~20 hydrogen bonds and five salt bridges (Figure 5d). Compared to other homologues, *C*LsoDHDPS has considerably more contacts (hydrogen bonds and salt bridges) at this interface and it buries a ~10% larger surface area from solvent.

**TABLE 4:** Comparison of the tight‐dimer and weak‐dimer interfaces of *C*LsoDHDPS (as shown in Figure 5c) and other characterized DHDPS enzymes using PISA (Krissinel & Henrick, 2007).

Organism	Ligand	PDB ID	Tight‐dimer interface (monomers *a*/*b*, *c*/*d*)			Weak‐dimer interface (monomers *a*/*c*, *b*/*d*)		
			BSA (Å^2^)	Hydrogen bonds	Salt bridges	BSA (Å^2^)	Hydrogen bonds	Salt bridges
*Ca*. L. solanacearum	Pyruvate	7LOY	1520	17	5	943	7	6
	Pyruvate/SSA	8GEK	1485	18	5	912	5	6
	Lysine	7LVL	1556	23	4	923	5	6
*E. coli*	Apo	1YXC	1289	9	‐	497	6	‐
	Pyruvate	3DU0	1226	9	‐	478	4	‐
	Pyruvate/SSA	4EOU	1318	10	‐	498	4	‐
	Lysine	1YXD	1297	9	‐	488	7	‐
*A. tumefaciens*	Apo	4I7U	1399	15	4	920	11	9
	Pyruvate	4I7V	1394	15	4	950	14	15
*B. anthracis*	Apo	1XL9	1393	10	‐	872	7	4
	Pyruvate	3HIJ	1392	10	‐	872	7	4
*T. maritima*	Apo	3PB2	1384	9	‐	878	20	24

*Note*: Succinic semi‐aldehyde is abbreviated to SSA and buried surface area is abbreviated to BSA.

The weak dimer interface between monomers *a*/*c* (and *b*/*d*) includes residues Ile168, Glu169, Val197, and His198 that connect the two dimers (Figure 5e). Compared to the tight‐dimer interface, the weak‐dimer interface has on average six hydrogen bonds and six salt bridges. This is increased compared to *Ec*DHDPS, but consistent with other bacteria DHDPS enzymes (Table 4). The weak‐dimer interface has been proposed to be a target for DHDPS inhibitor design (Mitsakos et al., 2008), since mutational studies of *E. coli* and *B. anthracis* DHDPS demonstrate that the formation of the tetramer is essential for enzyme activity (Griffin et al., 2008; Voss et al., 2010). Further, the weak‐dimer interface is a region of low conservation across bacterial species. For *C*LsoDHDPS, the residues comprising the weak‐dimer interface are similar to *A. tumefaciens*, but different to those in *E. coli*.

Although we were unable to crystalize the apo‐*C*LsoDHDPS, we can overlay and compare the solution small‐angle X‐ray scattering data for *C*LsoDHDPS with and without various combinations of ligands to test for conformational changes upon ligand binding. Four data sets were collected and compared: apo‐*C*LsoDHDPS, *C*LsoDHDPS + pyruvate, *C*LsoDHDPS + pyruvate/succinic semi‐aldehyde, and *C*LsoDHDPS + lysine (Figure 5f and collection/analysis statistics in Table Siii). The scattering data were collected to a high S range (0.005–0.5 S), meaning we can reasonably expect to detect even small deviations in the global structure. Since the scattering plots overlay very closely, we conclude that *C*LsoDHDPS does not change in structure upon substrate binding, consistent with the crystal structures. That there was little change in *C*LsoDHDPS structure when the allosteric inhibitor lysine is bound, suggests that its mechanism of allosteric inhibition must require only subtle structural changes (if any at all), which is also found for *Ec*DHDPS (Blickling & Knäblein, 1997; Blickling, Renner, et al., 1997; Dobson et al., 2005), but in contrast to the allosteric mechanism found in *N. sylvestris* DHDPS (Blickling, Beisel, et al., 1997). In the sections that follow, we will also demonstrate that the small‐angle X‐ray scattering data and the crystal structures match closely, evidence that the crystal structures faithfully represent the solution structures and are not affected by the crystal packing artifacts.

Overall, we demonstrate that the oligomeric state of *C*LsoDHDPS is tetrameric, as is the case for most other bacterial homologues. However, the interfaces between the monomers in *C*LsoDHDPS have increased surface area with more contacts, particularly across the functionally important tight‐dimer interface. Further, we demonstrate that ligand binding does not alter the oligomeric structure in any appreciable way. This suggests that any destabilizing effects of mutations accumulated as a result genetic drift is compensated for by increasing surface area and interactions at the tetrameric interfaces, a phenomenon suggested for enzymes that function at high temperatures (Reed et al., 2013; Vieille & Zeikus, 2001).

### 2.6 The CLsoDHDPS lysine and pyruvate binding sites are structurally the same as other DHDPS homologues

To explain the contrasting kinetic properties of *C*LsoDHDPS compared to homologues (i.e., ternary‐complex kinetic mechanism, slow turnover, and high affinity for its substrates), we undertook a comparative analysis of residues that form the active and allosteric sites. We hypothesized that structural rearrangements at these sites could account for these observations. A close examination of the *C*LsoDHDPS protein sequence, however, reveals that the residues within the allosteric and active site residues are largely retained (Figure Siii).

**FIGURE 6:**
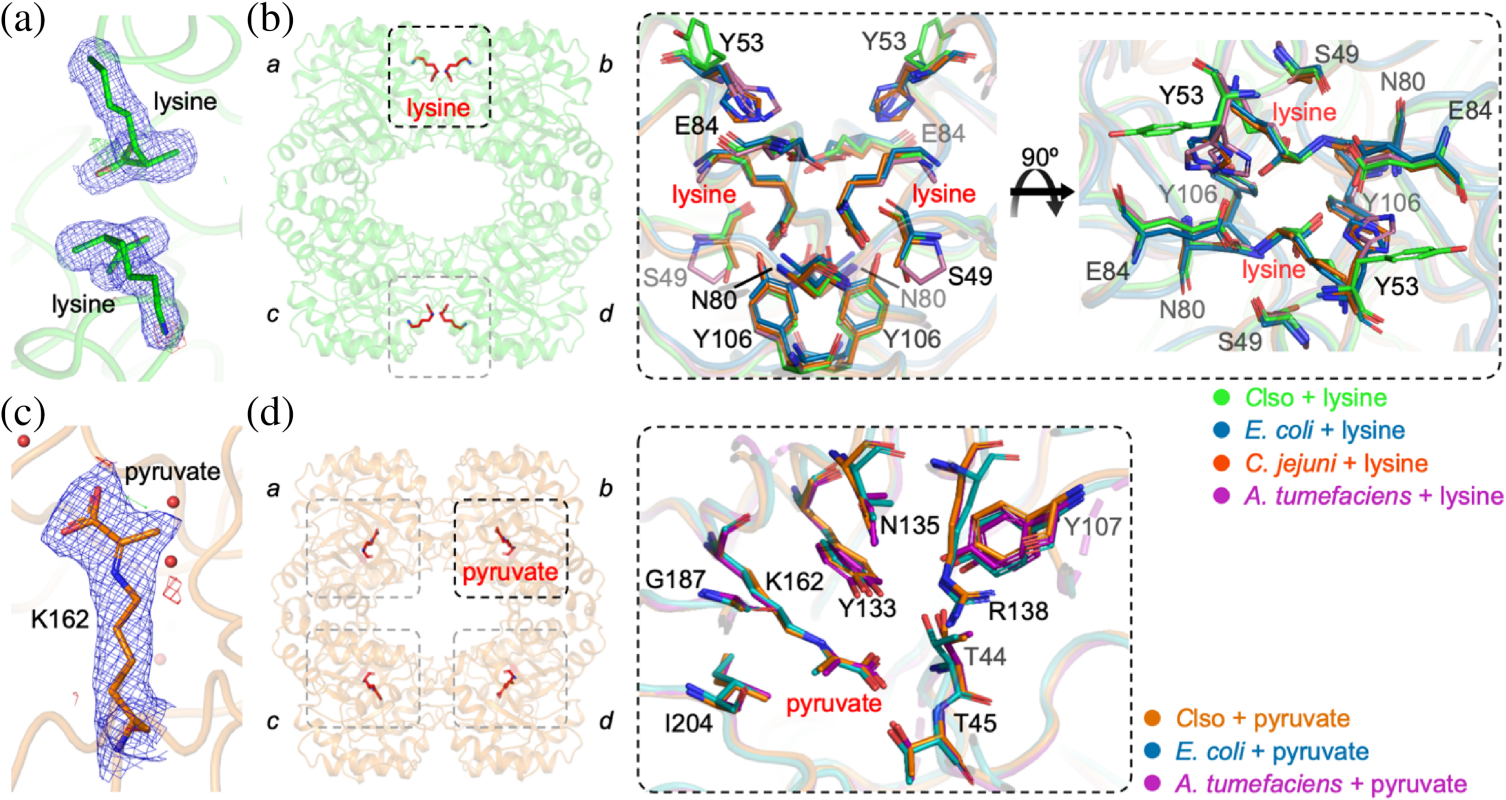
Structures of *C*LsoDHDPS with lysine or pyruvate. (a) An electron density map [2*F*_o_ − *F*_c_ at 1*σ* (blue), *F*_o_ − *F*_c_ at 3*σ* (green) and −3*σ* (red)] showing two lysine molecules within the cavity that forms the allosteric binding site. (b) The *C*LsoDHDPS tetramer showing lysine (red sticks) bound in the allosteric site at the tight‐binding interface formed through monomers *a*/*b* (or *c*/*d*). An enlargement of the allosteric site highlights residues that contact lysine, including Y53, E84, N80, Y106, which are within 4 Å. *C*LsoDHDPS with lysine (green, PDB ID: 7LVL) aligned with DHDPS homologues from *E. coli* (blue, PDB ID: 1YXD), *C. jejuni* (red, PDB ID: 4M19), and *A. tumefaciens* (magenta, PDB ID: 4I7W). In other weakly inhibited DHDPS homologues, Y53 is replaced with a histidine. (c) Pyruvate covalently bound to K162 within the active site. Several water molecules are also present close to the K162‐pyruvate adduct. (d) The *C*LsoDHDPS tetramer showing pyruvate (red sticks) bound within the active site of each monomer. An enlargement and alignment of the catalytic site residues for *C*LsoDHDPS with pyruvate bound to K162 (orange, PDB ID: 7LVL) aligned with equivalent structures from *E. coli* (blue, PDB ID: 3DU0), and *A. tumefaciens* (magenta, PDB ID: 4I7V) demonstrate highly conserved contacts for this substrate.

In plants and many bacteria, DHDPS catalyzes the first committed step towards lysine biosynthesis (branchpoint reaction) and is feedback regulated by the allosteric binding of lysine, the pathway product. We have demonstrated that *C*LsoDHDPS is weakly inhibited by lysine (Figure Siv), similar to homologues from other Gram‐negative bacteria. To determine how lysine binds to the allosteric cavity, which is ~15 Å away from the active site, we solved the crystal structure with bound lysine to 2.01 Å resolution. The presence and orientation of two lysine molecules in each of the allosteric sites was confirmed from an omit map (Figure Sva) and the refined (2*F*_o_ − *F*_c_) density allowed accurate modeling of the binding pose (Figure 6a). Small angle X‐ray scattering data confirms that the crystal structure faithfully represents the solutions structure (Figure Svb). An overlay with other Gram‐negative bacterial DHDPS homologues that are also weakly inhibited by lysine (*E. coli*, *A. tumefaciens*, and *C. jejuni*, Figure 6b) shows a highly conserved lysine binding pocket and the two lysine molecules engaging the site in an identical way, which is consistent with the similar low micromolar affinity for lysine. Although a definitive mechanism of allosteric inhibition has not been proposed for these homologues, it is likely that *C*LsoDHDPS uses an analogous mechanism.

Next, we solved the structure of *C*LsoDHDPS with the substrate pyruvate to 2.40 Å resolution. An omit map analysis (Figure Svc) confirms that pyruvate is within the active site close to Lys162 and small angle X‐ray scattering data confirm that the crystal structure faithfully represents the solutions structure (Figure Svd). The refined structure reveals continuous (2*F*_o_ − *F*_c_) electron density between Lys162 and pyruvate demonstrating that the enamine has formed (Figure 6c). The carboxylate group of pyruvate hydrogen bonds the side‐chain hydroxyl of Thr45 and the mainchain nitrogen atoms of Thr44 and Thr45 (Figure 6d). Pyruvate is positioned in a parallel plane to Tyr133 with the tyrosine hydroxyl group sitting above the enamine and Tyr133 is therefore well positioned to assist the formation of the Schiff base and subsequent enamine. The pyruvate methyl group points out from the active site, which places the enamine tautomer in an ideal position to attack the aldehyde of the incoming *S*‐aspartate‐β‐semialdehyde substrate. An overlay with homologous structures with pyruvate reveals highly similar active site architecture (Figure 6d) making it difficult to explain the decreased *K*_M_ for pyruvate (increased affinity). However, it is likely that the mechanism for the formation of the enamine is similar to other DHDPS enzymes. Lastly, we proposed above that the ternary‐complex mechanism had ordered substrate binding (pyruvate binding followed by *S*‐aspartate‐β‐semialdehyde binding). That we find pyruvate bound to the active site in the enamine form is consistent with an ordered substrate binding ternary‐complex mechanism.

Together, these structures demonstrate that *C*LsoDHDPS retains the key residues for both allosteric inhibition by lysine and catalytic activity. Neither lysine nor pyruvate binding alter the structure in any appreciable way and the binding sites and binding poses of lysine and pyruvate are highly similar to other DHDPS homologues. From these data alone, it is difficult to rationalize the differences in function of *C*LsoDHDPS compared with other DHDPS homologues.

### 2.7 CLsoDHDPS with bound pyruvate + succinic semi‐aldehyde reveals a different product

We then determined the 1.93 Å resolution structure of *C*LsoDHDPS with bound pyruvate + succinic semi‐aldehyde, which is an analogue of the second substrate *S*‐aspartate‐β‐semialdehyde in that it lacks the primary amine. The rationale for this experiment is that since there are limited changes in the structure of *C*LsoDHDPS with pyruvate or lysine, perhaps the explanation for the unusual kinetic behaviors stems from the enzyme interaction with and catalysis of the second substrate *S*‐aspartate‐β‐semialdehyde. Here, we expect the nucleophilic enamine (Schiff base) adduct of Lys162‐pyruvate to attack the electrophilic carbon of the aldehyde of succinic semi‐aldehyde to form a carbon–carbon covalent bond, as would occur with the natural substrate *S*‐aspartate‐β‐semialdehyde—this occurs *in crystal* for the *Ec*DHDPS (Blickling, Renner, et al., 1997; Boughton et al., 2012). An omit map analysis confirms the presence of density consistent with pyruvate and succinic semi‐aldehyde close to Lys162 (Figure Sve) and again small angle X‐ray scattering data confirms that the crystal structure faithfully represents the solutions structure (Figure Svf).

**FIGURE 7:**
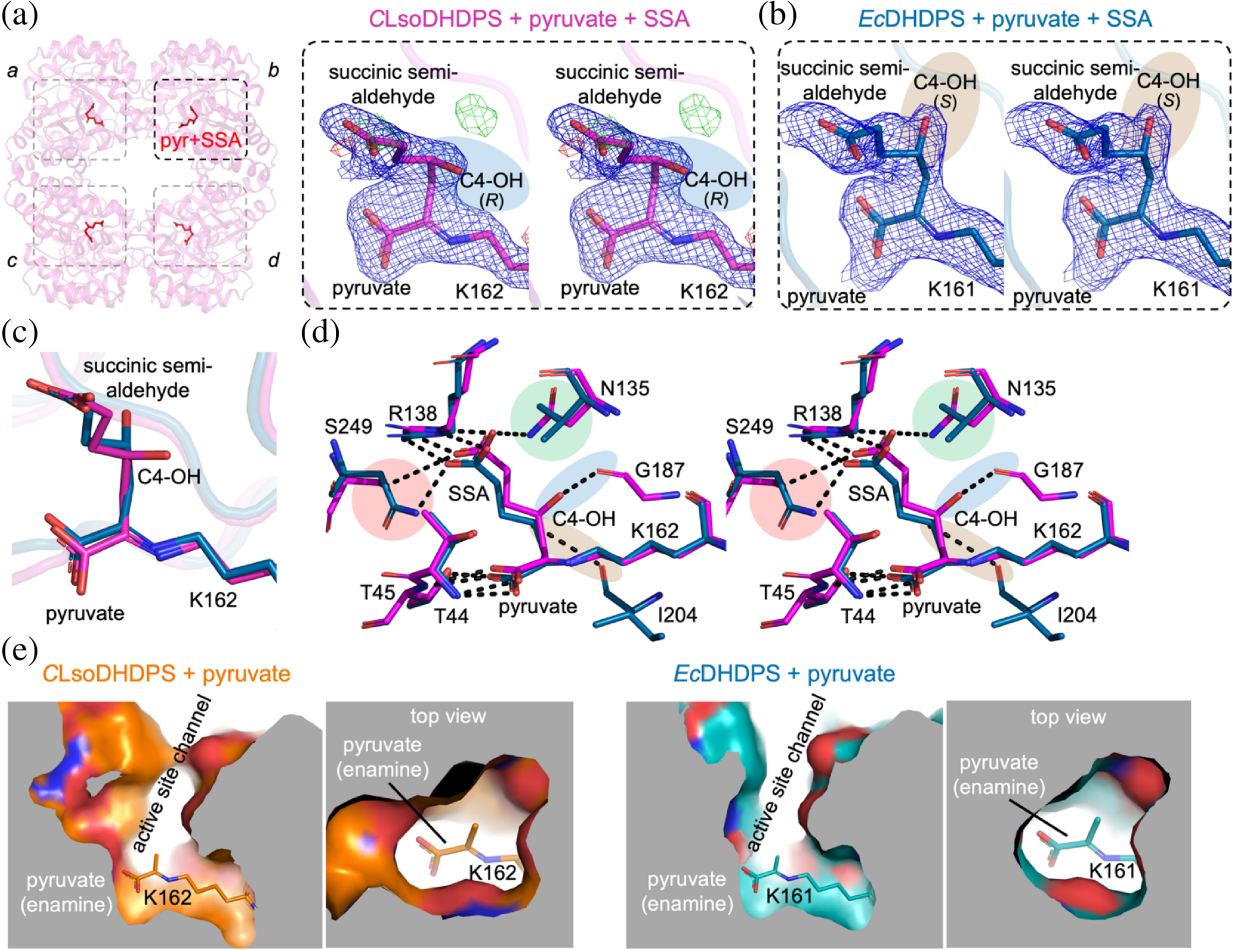
Structure of pyruvate + succinic semi‐aldehyde bound *C*LsoDHDPS. (a) and (b) Cross‐eyed stereo views comparing the refined electron density maps for the pyruvate + succinic semi‐aldehyde bound *C*LsoDHDPS and *Ec*DHDPS active sites, demonstrating the different stereochemical configurations of the C4‐hydroxyl (C4‐OH, highlighted with blue shading in *C*LsoDHDPS and brown shading in *Ec*DHDPS). The *σ* values for the electron density maps are as follows: the 2*F*_o_ − *F*_c_ map at 1*σ* (blue) and the *F*_o_ − *F*_c_ at 3*σ* (green) and −3*σ* (red). (c) A focus on the covalently bound ligands (pyruvate and succinic semi‐aldehyde) emphasizing the difference in stereochemistry within the active site. Residue numbering as per *C*LsoDHDPS. (d) An alignment and comparison of the active sites of *C*LsoDHDPS (magenta) and *Ec*DHDPS (blue, PDB ID: 4EOU) with pyruvate + succinic semialdehyde adducts covalently bonded to the catalytic lysine in different stereochemical configurations. Residue numbering as per *C*LsoDHDPS. (e) Cutaway views of the *C*LsoDHDPS and *Ec*DHDPS active sites with pyruvate, demonstrating the larger, more open volume that facilitates *S*‐aspartate‐β‐semialdehyde binding in for *C*LsoDHDPS. The orientations for the *C*LsoDHDPS and *Ec*DHDPS active sites, relative to the pyruvate/lysine adduct, are identical, as is the slab size and position.

Examination of the refined 2*F*_o_ − *F*_c_ electron density map (Figure 7a) shows continuous density between Lys162, pyruvate, and succinic semi‐aldehyde such that the carbon–carbon bond between these substrates has formed. Again, this is consistent with an ordered substrate binding ternary‐complex mechanism, where pyruvate binds first. There is clear density for the hydroxyl at the C4 position. The resolution of the data is sufficient to unambiguously model the hydroxyl in the *R*‐configuration. In contrast, analogous structures of *Ec*DHDPS + pyruvate + succinic semi‐aldehyde from separate research groups demonstrate that this hydroxyl is in the opposite *S*‐configuration (Blickling, Renner, et al., 1997; Boughton et al., 2012). On comparing the refined 2*F*_o_ − *F*_c_ electron density maps of *C*LsoDHDPS (Figure 7a) with pyruvate + succinic semi‐aldehyde with the analogous structure of *Ec*DHDPS (Figure 7b), it is clear that the change in stereochemistry at this position results in the C4 hydroxyl sitting in a different position and pointing in a different direction. This is more obvious when we overlayed the active sites for both structures (Figure 7c). It is this difference that provides insight into the altered kinetics properties.

When comparing the interactions that the substrates make in the active site (Figure 7d), the hydroxyl group at the C4 position in the *Ec*DHDPS structure forms a polar contact with the main‐chain of Ile203 (3.2 Å, highlighted with brown shading). In contrast, the altered stereochemistry of the C4‐hydroxyl in *C*LsoDHDPS results in a close polar contact with the main chain carbonyl of Gly187 instead (~2.76 Å, averaged over all monomers in the asymmetric unit, blue shading), and no connection to the equivalent Ile206 (~4.6 Å distant, averaged over all monomers in the asymmetric unit). The difference in stereochemistry must be a result of the aldehyde from the second substrate *S*‐aspartate‐β‐semialdehyde engaging the active site in a different conformation. That is, compared to the mechanism that operates in the *Ec*DHDPS homologue, the aldehyde is positioned such that nucleophilic attack must be from the opposite side of the carbonyl, resulting in the opposite stereochemical configuration.

This has several mechanistic consequences that explain the altered kinetics and stereochemistry of the product.

**FIGURE 8:**
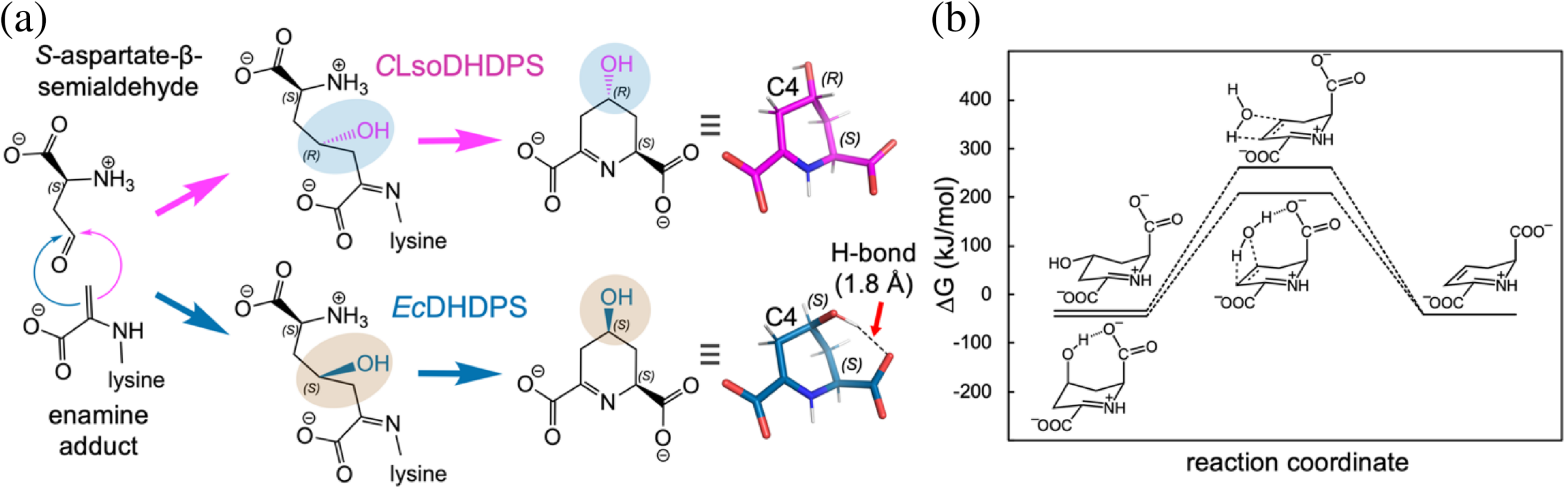
Reaction and energetics. (a) The reaction pathways that generate the different stereochemical configurations of the product (4*R*/*S*)‐hydroxy‐2,3,4,5‐tetrahydro‐(2*S*)‐dipicolinate. The C4‐OH is colored and shaded as in Figure 7a,b. To the right of the pane are the lowest energy conformers for each product, which reveals that only the (4*S*,2*S*)‐configuration can form a hydrogen bond between the C4‐OH and the C1‐COO^−^, which preferentially stabilizes this isomer by ~12 kJ/mol [based on computational benchmarking of analogous systems (Řezáč, 2020)]. (b) Reaction coordinate diagram for the solution‐phase dehydration of (4 *R* )‐hydroxy‐2,3,4,5‐tetrahydro‐(2*S*)‐dipicolinate (*C*LsoDHDPS, top pathway) and (4 *S* )‐hydroxy‐2,3,4,5‐tetrahydro‐(2*S*)‐dipicolinate (*E*cDHDPS, bottom pathway) to form (*S*)‐2,3‐dihydropicolinate. All free energies are computed relative to the substrates of the DHDPS‐catalyzed reaction, (*S*)‐aspartate‐β‐semialdehyde and pyruvate. The activation energy barrier (Δ_act_ *G*°) for dehydration of (4*S*)‐hydroxy‐2,3,4,5‐tetrahydro‐(2*S*)‐dipicolinate (*E*cDHDPS) is ~130 kJ/mol, but for (4*R*)‐hydroxy‐2,3,4,5‐tetrahydro‐(2*S*)‐dipicolinate (*C*LsoDHDPS) it is much higher at ~175 kJ/mol.

1. Differences in residues that bind *S*‐aspartate‐β‐semialdehyde are consistent with an increased affinity (decreased *K*_M_) for this substrate, particularly the carboxyl and amine moieties that position the aldehyde prior to condensation with pyruvate (Figure Svi). For both *C*LsoDHDPS and *E*cDHDPS, the carboxyl of succinic acid interacts closely with Arg138 (O—N = ~2.8 Å) and this residue is strictly conserved in DHDPS enzymes (Figure Siii). However, Asn135 and Ser249 in *C*LsoDHDPS are equivalent to Val135 and Asn248 in *E*cDHDPS (Figure 7d), which are also highly conserved residues across DHDPS enzymes, except for *Candidatus* Liberibacter species (Figure Siii). In *Ec*DHDPS the carboxyl of succinic acid contacts Asn248 (O—N = 2.7 Å), but in *C*LsoDHDPS it contacts both Ser249 (O—O = ~4.2 Å) and Asn135 (O—N = 2.9 Å); this additional hydrogen bond is consistent with the increased affinity (decreased *K*_M_) for *S*‐aspartate‐β‐semialdehyde.
2. The difference in succinic semialdehyde binding and larger active site explains the difference in the stereochemistry of the product. Because the pyruvate enamine structures are the same across all DHDPS homologues, including *C*LsoDHDPS (Figure 6d), it follows that *S*‐aspartate‐β‐semialdehyde must bind the active site differently in order to present the aldehyde (electrophile) in a different orientation to provide the (*R,S*)‐isomeric product. The alternate binding pattern places succinic acid in a slightly different position in the active site, presumably also placing the aldehyde in a different position prior to carbon–carbon bond formation, resulting in the different stereochemical outcome (Figure Svi). Comparing the volume and shape of the active sites between *C*LsoDHDPS and *Ec*DHDPS (Figure 7e) reveals that the active site cavity for *C*LsoDHDPS is significantly wider at the *S*‐aspartate‐β‐semialdehyde binding site, allowing *S*‐aspartate‐β‐semialdehyde more conformational flexibility within the *C*LsoDHDPS active site, again consistent with the alternate stereochemical outcome of the product. A recent review concluded that active site linked tunnels and channels in enzymes affect activity, specificity, promiscuity, enantioselectivity, and stability (Kokkonen et al., 2019). The wider active site channel of *C*LsoDHDPS could also allow faster and more efficient passage of substrates into and products out of the active site, consistent with the increased affinity for the substrates.
3. There are two reasons why tighter *S*‐aspartate‐β‐semialdehyde binding in the active site may lead to decreased *k*_cat_—a trade‐off. Firstly, the condensation reaction between the pyruvate enamine and *S*‐aspartate‐β‐semialdehyde is thought to be the rate determining step in the catalytic mechanism because it involves carbon–carbon bond formation. In *Ec*DHDPS, Ile203 (*Ec*DHDPS numbering) binds the hydroxyl of the pyruvate‐*S*‐aspartate‐β‐semialdehyde adduct, given that high resolution structures show it binds the hydroxyl of substrate analogues hydroxypyruvate (Dobson et al., 2008) and pyruvate succinic semialdehyde (Boughton et al., 2012), and is proposed to play a key role in this step. In *C*LsoDHDPS, however, the C4‐hydroxyl binds Gly186 and not Ile206 (*C*LsoDHDPS numbering) meaning that the isoleucine main chain does not participate during catalysis. Secondly, the final step of catalysis is the cyclization through *S*‐aspartate‐β‐semialdehyde amine. If the *S*‐aspartate‐β‐semialdehyde carboxyl is constrained by additional bonding to Asn135 and Ser249, as seen in the *C*LsoDHDPS succinic acid bound structure, then the cyclization step may be slowed. These provide mechanistic explanations for the trade‐off between *k*_cat_ and *K*_M_^ASA^.
4. Next, we considered the energetic consequences of the different products, which provides a surprising insight into the evolution of DHDPS enzymes more broadly. Quantum chemical modeling reveals that (4*S*)‐hydroxy‐2,3,4,5‐tetrahydro‐(2*S*)‐dipicolinate can form an intramolecular hydrogen bond that preferentially stabilizes this conformer by ~12 kJ/mol compared to the (4*R*)‐hydroxy‐2,3,4,5‐tetrahydro‐(2*S*)‐dipicolinate isomer produced by *C*LsoDHDPS (Figure 8a). A fundamental feature of enzymes is that they do not affect the equilibrium of the reaction (energy of the substrates vs. the products), but instead catalyze the rate at which equilibrium is achieved (decrease the energy of the transition state) (Cornish‐Bowden, 1995; Fersht, 1999). Since the product is both structurally and energetically different and therefore the equilibrium for this step has changed, evolution has in this case fashioned a new enzyme.
5. The intramolecular hydrogen bond present in the (*S,S*)‐isomer, but not the (*R,S*)‐isomer, also acts as a catalyst for the subsequent solution‐phase dehydration reaction, providing a lower‐energy pathway (decreased transition state energy) to the (*S*)‐2,3‐dihydropicolinate product (Figure 8b). This means that the subsequent dehydration step in the pathway is also changed, as it too will be slower compared to other bacterial diaminopimelate pathways, where the intramolecular hydrogen bond catalyzes dehydration. Given that the product of the spontaneous dehydration is the same, the pathway likely continues to diaminopimelate and lysine as in other bacteria.

To summarize, our high‐resolution structure of *C*LsoDHDPS with bound pyruvate + succinic semi‐aldehyde (1.93 Å) demonstrates an alternative stereochemistry for the product in the active site. This is driven by subtle changes in the *S*‐aspartate‐β‐semialdehyde binding site that explains the increase affinity for the substrate, a lower turnover, and for a different stereochemical product. The (*R,S*)‐isomer product of *C*LsoDHDPS is energetically less stable and has a higher energy for dehydration compared to the (*S,S*)‐isomer product of other DHDPS enzymes, representing a fundamental change in the chemistry.

## 3 DISCUSSION

Our objective was to define whether genome reduction and population bottlenecks through vertical transmission influence enzyme structure and function, as predicted. Such studies are rare because proteins from reduced genomes tend to be highly unstable and therefore challenging to work with. To date, there are no protein structures for *Ca*. L. solanacearum reported and structures from Liberibacter species in general are limited (Jiang et al., 2014; Kumar, Dalal, et al., 2019; Kumar, Kesari, et al., 2019; Nan et al., 2020; Saini et al., 2018), which hinders our understanding of the biology of this genus. We studied the *Ca*. L. solanacearum lysine DHDPS, which is well defined in other bacterial systems and provides a unique opportunity to assess how evolution shapes enzymes in context of a reduced genome and a bottlenecked lifestyle.

It is difficult to untangle the effects of natural selection (horizontal transmission via the plant) and drift (bottlenecking during successive vertical transmission) given the complex lifestyles of *Ca*. L. solanacearum. Yet, the characteristics of the *Ca*. L. solanacearum genome are similar to bacteria such as *B. aphidicola* in that there is a high AT content and reduced size. Consistent with the proposal that genome reduction and population bottlenecks destabilize protein structure (van Ham et al., 2003), we demonstrate that *C*LsoDHDPS is destabilized relative to homologues, particularly in the presence of ligands. Moreover, the aggregation propensity of *C*LsoDHDPS is increased compared to homologues from bacteria with significantly larger genomes that are expected to evolve through natural selection. Indeed, this trend is also seen across other enzymes in the lysine biosynthetic pathway.

Our data supports the hypothesis that increased chaperone activity counters protein instability resulting from the accumulation of deleterious mutations. In addition, for *C*LsoDHDPS at least, instability could be partly overcome through increasing both the buried surface areas in oligomeric interfaces and the number of hydrogen bonds and salt bridges, an adaptation akin to that found in thermophilic enzymes that operate at high temperatures. Comparing *C*LsoDHDPS with the thermophilic homologue from *T. maritima*, demonstrates that the catalytically important tight‐dimer interface, which comprises residues that form the active site and the allosteric binding site, is significantly stabilized, whereas in *Tm*DHDPS it is the weak‐dimer interface that has increased stability. This perhaps reflects a need for *Tm*DHDPS when operating at high temperatures to retain its tetrameric state, which has been demonstrated to be important for function though minimizing the protein dynamics through the dimer (and therefore the active sites). In contrast, *C*LsoDHDPS has stabilized the tight‐binding interface that could compensate for destabilizing mutations that may occur due to drift.

Surprisingly, *C*LsoDHDPS is mechanistically the most divergent of all DHDPS enzymes studied to date. It binds its substrates with significantly higher affinity (low *K*_M_) yet is comparatively slow (low turnover); that is, there appears to be a trade‐off between an increased binding affinity for the substrates and decreased catalysis. The structures of *C*LsoDHDPS presented here suggest the molecular basis for this trade‐off. The subtle differences in the active site suggest that *S*‐aspartate‐β‐semialdehyde binds differently and employs alternative catalytic residues, leading to increased affinity for the substrates, a different rate determining step and a different product.

Given that *C*LsoDHDPS catalyzes a reaction that results in a different product [(4*R*)‐hydroxy‐2,3,4,5‐tetrahydro‐(2*S*)‐dipicolinate] that is energetically different (meaning the equilibrium for catalysis is different), it is in fact a new enzyme. Detailed structural data provides a molecular basis for these differences, whereby subtle differences in the active site affect binding and catalysis. We suggest that bacteria with endosymbiotic lifestyles present a rich source of interesting and variable enzymes useful for understanding enzyme function and/or informing protein engineering efforts.

The mechanistic differences of *C*LsoDHDPS provide a new perspective to understand how DHDPS enzymes may have evolved in organisms expected to be under stringent natural selection. Specifically, these DHDPS enzymes likely evolved to afford the product (4*S*)‐hydroxy‐2,3,4,5‐tetrahydro‐(2*S*)‐dipicolinate, because it is more stable and able to catalyze the dehydration of the hydroxyl to form the substrate for the next enzyme in the pathway. In contrast, although the (4*R*)‐hydroxyl product is destabilized and cannot catalyze the dehydration in solution, the trade‐off may be an active site that binds its substrate with higher affinity, which in the context of niches where *C*LsoDHDPS functions (plant phloem and insect gut) may be an advantage.

We see two future directions triggered by our work. Firstly, to study the effect on drift on enzymes requires a context where drift dominates. Our next study will focus on DHDPS enzymes from *W. pipientis*, *B. aphidicola*, and *Ca*. Carsonella ruddii to help elucidate the effect of drift on these homologues. Secondly, *Ca*. L. solanacearum has significant economic impact on the potato industries in Central and North America, and New Zealand. Novel disease management strategies will require an understanding of the structure and function of essential (target) proteins required for pathogen survival to assist in the design of antimicrobials against this plant pathogen. Our kinetic and structural studies demonstrate that *C*LsoDHDPS is the most unusual of DHDPS enzyme studied to date. These differences, including kinetic mechanism, structure of the active site and substrate binding, could be exploited to develop specific inhibitors of *C*LsoDHDPS activity, but not of the corresponding enzyme in the plants they infect.

## 4 EXPERIMENTAL

### 4.1 Materials

The substrate *S*‐aspartate‐β‐semialdehyde was synthesized using the previous methods (Roberts et al., 2003). The *E. coli* homologue of dihydrodipicolinate reductase was used in the coupled kinetic assay and was prepared using reported methods (Dobson, Valegård, et al., 2004).

### 4.2 Gene expression analysis

#### 4.2.1 Primer design

Primers were designed using the National Centre for Biotechnology Information (NCBI) Primer‐Basic Local Alignment Search Tool (Primer‐BLAST) (Ye et al., 2012). The genes and the primers used to amplify the gene products are described in Tables Sv and Svi. Infected and uninfected psyllid and plant samples were tested for each primer pair in ensure primer specificity.

#### 4.2.2 DNA extraction

Adult tomato potato psyllids were collected from *C*Lso‐infected colonies. DNA was extracted from five psyllids from each colony using a cetyl trimethylammonium bromide extraction method (Beard & Scott, 2013). DNA was also extracted from infected tomato plants using the cetyl trimethylammonium bromide extraction method (Beard et al., 2013). A semi‐nested qPCR protocol (Beard & Scott, 2013) was used to determine the titer of *Ca*. L. solanacearum present in DNA extracted from infected psyllids or tomato plants.

#### 4.2.3 RNA extraction and cDNA synthesis

Gene expression experiments were performed using tomato plants, *Solanum lycopersicum* L., and psyllids infected with *Ca*. L. solanacearum. Prior to RNA extraction, plant samples were stored in RNA*later*™. The Geneaid Total RNA Mini Kit and the Aurum™ total RNA fatty and fibrous tissue kit were used for RNA extraction from psyllid and plant samples, respectively, as described by the manufacturer's instructions. The isolated RNA was treated with RQ1 RNase‐free DNase (Promega, Madison, WI) as described by the manufacturer. RNA was amplified using primers (Tables Sv and Svi) by conventional PCR to test for the presence of any genomic DNA. cDNA was synthesized from 1 μg of total RNA using the iScript™ cDNA Synthesis Kit (BioRad) as described by the manufacturer.

#### 4.2.4 Reverse transcription quantitative PCR

All RT‐qdPCR reactions were performed on a StepOne Plus™ real time PCR system (Thermo Fisher Scientific). SsoAdvanced™ universal SYBR reagent was mixed with appropriate primers at a final concentration of 0.3 μM and 5 μL cDNA in a reaction with a total volume of 20 μL. All samples were run in triplicate. Recombinase A (*recA*, KJZ80672) and the DNA‐directed RNA polymerase beta subunit (*rpb*, KJZ81364.1) were used as reference genes to provide normalization of relative quantification for the target gene.

#### 4.2.5 Data analysis

The relative quantification of gene expression level was determined by the comparative C_T_ method 2^−ΔΔCT^ (Livak & Schmittgen, 2001), where ^ΔΔ^C_T_ is calculated by the following equation:

$$\Delta \Delta C_{T}=\left({C_{T}}^{\text{target}}–{C_{T}}^{\text{reference}}\right)\mathrm{Ca}.L.\text{solanacearum infected}–\left({C_{T}}^{\text{target}}–{C_{T}}^{\text{reference}}\right)\;\text{control}$$

Standard deviation and *p*‐values were calculated using a Student's *t*‐test (Yuan et al., 2006).

### 4.3 Aggregation propensity

The AGGRESCAN algorithm (Conchillo‐Solé et al., 2007) was used to predict the aggregation propensity of proteins in this study (http://bioinf.uab.es/aggrescan/). Amino acid sequences were submitted to AGGRESCAN in FASTA format, and Na^4^vSS (Normalized a^4^v Sequence Sum for 100 residues) values were selected to compare the predictions.

### 4.4 Protein expression and purification

*C*LsoDHDPS and *C*Lso pyruvate kinase (PykF) were purified by the method previously described (Gilkes et al., 2019). Briefly, *E. coli* BL21 that harbored both the pGRO7 plasmid for GroEL and GroES expression as well as the pET24_*C*Lso_*dapA* plasmid, were grown overnight at 37°C. Overnight cultures were used to inoculate 1 L of Luria‐Bertani broth and grown at 37°C with shaking to an OD_600_ = 0.2, at which point chaperone expression was induced with L‐arabinose at 0.5 mg/mL. The cultures were grown further to an OD_600_ of 0.5, at which point the protein of interest was induced with 1 mM IPTG and incubated at 16°C for 20 h with shaking. Protein concentration was measured by the Bradford method (Bradford, 1976). The protein was purified using a nickel affinity column (GE Healthcare Life Sciences) equilibrated with PBS buffer with 1 mM DTT, 5% glycerol, and 40 mM imidazole (pH 7.4). This was followed by size exclusion chromatography using a HiLoad 16/200 Superdex column (GE Healthcare Life Sciences). Unless otherwise stated, enzymes were manipulated at 4°C or on ice. *E. coli* DHDPS and pyruvate kinase were expressed and purified as previously described (Dobson, Griffin, et al., 2004; Zhu et al., 2010).

### 4.5 Differential scanning fluorimetry

The QuantStudio 3 real‐time PCR system (ThermoFisher Scientific) was used to record the thermal stability of the purified protein (*Ec*DHDPS, *C*LsoDHDPS, *Ec*PykF, and *C*LsoPykF) with and without potential substrates. Reactions consisted of 1 mg/mL protein, 5× SYPRO Orange (prepared as a 50× stock) and the 5–10 mM of the desired substrate in a reaction volume of 30 μL. Samples were heated from 4 to 95°C at a ramp rate of 0.05°C, taking fluorescent readings at each time point. Triplicate measurements were performed. To validate the experiment, one positive control using a well‐characterized melting curve (lysozyme at 1 mg), along with two negative controls (protein only and dye only) were conducted. Data analysis was performed using Protein Thermal Shift software (version 1.3, Applied Biosystems), where an apparent melting point (*T*_m_^D^) of each sample in°C was obtained from the highest point of the first derivative plot (Figure Si).

### 4.6 Thioflavin‐T aggregation assays

Aggregation of *E. coli* and C*Lso*DHDPS was measured in 96‐well half area non‐binding plates (Corning 3881) at a protein concentration of 33 μM with addition of 20 μM thioflavin‐T in phosphate buffered saline, pH 7.5. Samples were measured in triplicate and each had a final volume of 50 μL. The plate was sealed with a MicroAmp Optical Adhesive Film (Thermo Fisher Scientific) and measured on a Molecular Devices M5 microplate reader set to 37°C. Thioflavin‐T fluorescence was measured (ex/em 438/485 nm) at 2 min intervals with 90 s of linear shaking in between measurements in bottom read mode. Half‐times were fit using the Amylofit web server (Meisl et al., 2016).

### 4.7 Steady‐state enzyme kinetics

DHDPS activity was measured using a dihydrodipicolinate reductase coupled assay, as previously described (Dobson, Valegård, et al., 2004). The reaction was initiated with *S*‐aspartate‐β‐semialdehyde, after the cuvette had pre‐incubated at 30°C for 10 min. Assays were performed in HEPES buffer (100 mM at pH 8) at 30°C, kept constant using a circulating water bath. Care was taken to ensure that the enzymes and substrates were stable over the course of the assay. Care was also taken to ensure an excess of DHDPR; about 20 μg per assay was used. The *K*_M_ values for *C*LsoDHDPS were unknown; therefore, apparent *K*_M_ values were initially determined using a range of substrate concentrations. True kinetic constants (*K*_M_ and *k*_cat_) were determined using a range of concentrations approximately 10‐fold above and below these values. Initial velocities were reproducible within 10%. All data were analyzed using *R*. Data were fitted to the appropriate models as judged by the *R* ^2^ value and the Akaike information criterion (AIC).

### 4.8 Small angle X‐ray scattering

Small angle X‐ray scattering (SAXS) data were collected on the SAXS/WAXS beamline equipped with a Pilatus 1M detector (169 × 179 mm^2^, effective pixel size, 172 μm × 172 μm) at the Australian Synchrotron. A sample detector distance of 1600 mm was used, providing a *q* range of 0.006–0.5 Å^−1^. The purified protein was injected onto an inline Superdex S200 Increase 5/150 GL SEC column (GE Healthcare), equilibrated with 1× PBS buffer (pH 7.4) supplemented with 0.1% (w/v) sodium azide, at a flow rate of 0.35 mL/min. Co‐flow SAXS was used to minimize sample dilution and maximize signal to noise ratio. Scattering data were collected in 1 s exposures (*λ* = 1.0332 Å) over 500 frames, using a 1.5 mm glass capillary, at 12°C.

Analysis of the scattering data were performed using the ATSAS software package (version 2.8.4) (Franke et al., 2017). The molecular mass of the samples was estimated using the SAXS‐MoW2 package. 2D intensity plots were radially averaged, normalized to sample transmission, and background subtracted using CHROMIXS. Guinier plots were analyzed using PRIMUS (Franke et al., 2017) to assess data quality. Indirect Fourier transform of the data were performed using GNOM (Svergun, 1992) to generate the *P*(*r*) distribution. Theoretical scattering curves were generated from atomic coordinates and compared with experimental scattering curves using CRYSOL (Svergun et al., 1995). Data collection and analysis statistics are summarized in Table Siii.

### 4.9 X‐ray crystallography

Crystallization studies were performed using freshly purified protein, concentrated to 14 mg/mL. Crystallization trials used the sitting drop vapor diffusion method, at 20°C, with droplets consisting of 150 nL of protein solution and 150 nL of reservoir solution. Rectangular *C*LsoDHDPS crystals were produced using the JCSG+ Suite screen in condition A2 (20% w/v polyethylene glycol 3000, 0.1 M sodium citrate, pH 5.5) at 20°C. For ligand bound structures, the crystals were soaked for 7 days in mother liquor with either: 100 mM lysine, 100 mM pyruvate or 100 mM of both succinic semi‐aldehyde and pyruvate. Prior to X‐ray data collection, the crystals were soaked in cryoprotectant solution consisting of 85% reservoir solution and 15% glycerol–ethylene glycol (50:50 mix) prior to being flash frozen in liquid nitrogen.

Diffraction data were collected at 110 K on the MX2 beamline at the Australian Synchrotron (McPhillips et al., 2002) with the EIGER × 16M detector (*λ* = 0.9537 Å). All liganded crystals belong to the space group C2 with diffraction to 2.40 Å for *C*LsoDHDPS + pyruvate, 1.93 Å for *C*LsoDHDPS + pyruvate/SSA, and 2.01 Å for *C*LsoDHDPS + lysine (Table Siv). Diffraction data sets were processed and scaled using XDS and AIMLESS from the CCP4i2 program suite (Potterton et al., 2018). The resulting intensity data were analyzed using PHENIX XTRIAGE (Adams et al., 2010). Molecular replacement was performed using PHASER (McCoy et al., 2007) with *A. tumefaciens* DHDPS (PDB ID: 4I7U) as the search model. CHAINSAW (Stein, 2008) from the CCP4i2 suite was used to prepare the model of *C*LsoDHDPS, omitting waters and reducing it to its monomeric form. Structural refinement of the resulting six monomers was performed using REFMAC5 (Murshudov et al., 1997) with iterative model building using COOT (Emsley & Cowtan, 2004). Initial refinement initially applied non‐crystallographic restraints followed by simulated annealing using PHENIX with the structure validated using MolProbity (Chen et al., 2010). The occupancy for the ligands was checked using PHENIX.REFINE and were all close to 1.0. Images were generated in PyMOL (DeLano, 2002) or Chimera (Pettersen et al., 2021). Data collection and model‐refinement statistics are in Table Siv.

### 4.10 Computational modeling

Geometries of (4*S*)‐hydroxy‐2,3,4,5‐tetrahydro‐(2*S*)‐dipicolinate, (4*R*)‐hydroxy‐2,3,4,5‐tetrahydro‐(2*S*)‐dipicolinate, *S*‐aspartate‐β‐semialdehyde, pyruvate, and *S*‐2,3‐dihydropicolinate were optimized at B3LYP/6‐31G*, considering all possible rotamers. The lowest energy conformers identified were confirmed as minima by the absence of imaginary frequencies following harmonic frequency calculations, from which zero‐point (*H*_ZVPE_), enthalpic (*H*_therm_), and entropic (*S*_therm_) contributions to the free energy of each species under standard thermodynamic conditions were also obtained. Single point calculations were performed at ωB97X‐V/def2‐QZVPPD to more accurately estimate the electronic energy (*U*_elec_) and absolute free energies computed as:

$$G=U_{\text{elec}}\left(\mathrm{\omega B}9\text{7X}-V\right)+H_{\text{ZVPE}}\left(B3\mathrm{LYP}\right)+H_{\text{therm}}\left(B3\mathrm{LYP}\right)–{\mathrm{TS}}_{\text{therm}}\left(B3\mathrm{LYP}\right)$$

Free energies of reaction, Δ*G*, were computed relative to the free energy of (*S*)‐aspartate‐β‐semialdehyde and pyruvate in solution. Transition states for eliminative dehydration were identified by performing 2D relaxed scans along the C—O and O—H bond length coordinates (step size = 0.2 Å), followed by an automated transition state optimization. Frequency calculations were performed to confirm that these optimizations had converged a first‐order transition state, indicated by the presence of a single imaginary frequency. Transition state free energies were computed as above, excluding the imaginary frequency vibrational mode that corresponds to the reaction coordinate. All calculations were performed under the influence of a polarizable continuum solvation model with a dielectric constant chosen to resemble water (*ε* = 78.36), using the QChem5.3 programme package (Epifanovsky et al., 2021).

## AUTHOR CONTRIBUTIONS

**Jenna M. Gilkes:** Conceptualization; investigation; methodology; writing – original draft; writing – review and editing; formal analysis; data curation; project administration; visualization. **Rebekah A. Frampton:** Conceptualization; investigation; writing – review and editing; validation; methodology; formal analysis; supervision. **Amanda J. Board:** Investigation; methodology; formal analysis. **André O. Hudson:** Writing – review and editing; investigation; supervision; visualization. **Thomas G. Price:** Investigation; formal analysis; methodology. **Vanessa K. Morris:** Investigation; formal analysis; writing – review and editing. **Deborah L. Crittenden:** Investigation; conceptualization; validation; formal analysis; writing – review and editing; supervision. **Andrew C. Muscroft‐Taylor:** Conceptualization; funding acquisition; supervision. **Campbell R. Sheen:** Investigation; validation; supervision; formal analysis. **Grant R. Smith:** Conceptualization; investigation; funding acquisition; writing – review and editing; validation; methodology; formal analysis; supervision; resources; project administration. **Renwick C. J. Dobson:** Supervision; resources; investigation; conceptualization; funding acquisition; writing – review and editing; validation; formal analysis; project administration; methodology; visualization.

## Supporting information


**Appendix S1:** Supporting information.

## Data Availability

The data underpinning this article are available in the article and in its online supplementary material. Any further data required associated with this article will be shared on reasonable request to the corresponding author.
