# Supplementary material for: A new lysine biosynthetic enzyme from a bacterial endosymbiont shaped by genetic drift and genome reduction
Source: Protein Sci. 2024 Jun 26;33(7):e5083. doi: 10.1002/pro.5083 (PMC11201819; doi:10.1002/pro.5083)
Supplement: Supplementary file 1 — Appendix S1: Supporting information. [file PRO-33-e5083-s001.docx]

**Supplementary information for:**

A new lysine biosynthetic enzyme from a bacterial endosymbiont shaped by genetic drift and genome reduction.

Jenna M. Gilkes, Rebekah A. Frampton, Amanda Board, André O. Hudson, Thomas G. Price, Vanessa K. Morris, Deborah L. Crittenden, Andrew C. Muscroft-Taylor, Campbell R. Sheen, Grant R. Smith, Renwick C.J. Dobson

**Supplementary Tables**

**Supplementary Table i |** Comparison of thermal melting temperatures between *C*LsoDHDPS with pyruvate (which is likely to be free in the cell) and other bacterial homologues. A caveat for this comparison is that these were determined by different methods and conditions (buffers, pH, protein concentrations). * Represents in the absence of pyruvate.

| **Organism** | ***T*_M_ *+ pyruvate*** |
| --- | --- |
| *Ca*. L. solanacearum (this work) | 56 |
| *E. coli* (this work) | 65 |
| *M. tuberculosis* (Kefala et al. 2008) | ~82* |
| *T. maritima* (Pearce et al. 2006) | >80* |
| *S. pneumoniae* (Dogovski et al. 2013) | 72* |
| *B. anthracis* (Domigan et al. 2009) | ~70 |
| *N. meningitides* (Devenish et al. 2009) | 61.6 |
| *B. licheniformis* (Halling and Stahly 1976) | 84 |

**Supplementary Table ii |** Comparison of kinetic constants between *C*LsoDHDPS and other bacterial homologues.

| **Organism** | ***k_cat_* (s^-1^)** | ***K*_M_^pyruvate^ (mM)** | ***K*_M_^(^*^S^*^)-ASA^  (mM)** | ***k*_cat_/*K*_M_^pyruvate^  (M^-1^ s^-1^)** | ***k*_cat_/*K*_M_^(^*^S^*^)-ASA^  (M^-1^ s^-1^)** | ***k_s_*** |
| --- | --- | --- | --- | --- | --- | --- |
| *Ca*. L. solanacearum (this work) | 6 | 0.012 | 0.033 | 5.0 × 10^5^ | 1.8 × 10^5^ | 0.002 |
| *E. coli* (Karsten 1997) | 188 | 0.19 | 0.12 | 9.9 × 10^5^ | 1.6 × 10^6^ | - |
| *M. tuberculosis* (Kefala et al. 2008) | 138 | 0.17 | 0.43 | 8.1 × 10^5^ | 3.2 × 10^5^ | - |
| *A. thaliana* (Griffin et al. 2012) | 93 | 1.0 | 0.09 | 9.3 × 10^4^ | 1.0 × 10^6^ | - |
| *T. maritima* (Pearce et al. 2006) | 5 * | 0.053 | 0.16 | 9.5 × 10^4^ | 3.2 × 10^4^ | - |
| *S. pneumoniae* (Dogovski et al. 2013) | 22 | 2.6 | 0.044 | 8.6 × 10^3^ | 5.0 × 10^5^ | - |
| *B. anthracis* (Domigan et al. 2009) | 76 | 0.43 | 0.18 | 1.8 × 10^5^ | 4.2 × 10^5^ | - |
| *N. meningitides* (Devenish et al. 2009) | 47 | 0.50 | 0.052 | 9.4 × 10^4^ | 9.0 × 10^5^ | - |
| *V. cholerae* (Gupta et al. 2018) | 34 | 0.14 | 0.08 | 2.4 × 10^5^ | 4.3 × 10^5^ | - |
| *C. jejuni* (Skovpen and Palmer 2013) | 76 | 0.35 | 0.16 | 2.2 × 10^5^ | 4.8 × 10^5^ | - |
| *P. aeruginosa 1* (Impey et al. 2020) | 44 | 0.23 | 0.15 | 1.9 × 10^5^ | 2.9 × 10^5^ | - |
| *P. aeruginosa 2* (Impey et al. 2020) | 29 | 0.12 | 0.053 | 2.4 × 10^5^ | 5.5 × 10^5^ | - |

* Data collected at 20 °C, far below this enzyme’s native temperature (~80 °C)

**Supplementary Table iii |** Data analysis statistics and collection parameters for the small angle X-ray scattering (SAXS) experiments.

| **Data analysis** | **apo-DHDPS** | **DHDPS + pyruvate** | | | **DHDPS + pyruvate/SSA** | | **DHDPS + lysine** |
| --- | --- | --- | --- | --- | --- | --- | --- |
| *I*(0) (cm^-1^) (Guinier analysis) | 0.11 ± 0.0002 | 0.0840 ± 0.0001 | | | 0.11 ± 0.0001 | | 0.043 ± 0.0001 |
| R_g_ (Å) (Guinier analysis) | 31.08 ± 0.30 | 32.93 ± 0.21 | | | 32.8 ± 0.25 | | 31.5 ± 0.27 |
| *I*(0) (cm^-1^) (*P*(*r*) analysis) | 0.11 | 0.08 | | | 0.11 | | 0.04 |
| R_g_ (Å) (*P*(*r*) analysis) | 31.08 | 32.93 | | | 32.8 | | 31.5 |
| D_max_ (Å) | 89.4 | 89.8 | | | 90.5 | | 92.8 |
| Porod volume (Å^-3^) | 164 000 | 214 000 | | | 172 000 | | 171 000 |
| Molar mass (Porod volume, kDa) | 96 470 | 125 882 | | | 129 911 | | 100 588 |
| Molar mass (SAXSMoW2*, kDa) | 113 650 | 123 248 | | | 101 176 | | 124 742 |
| Tetrameric mass from sequence (kDa) | 132 000 | 132 000 | | | 132 000 | | 132 000 |
| **Data collection parameters** |  | |  |  | |  | |
| Instrument | Australian Synchrotron SAXS/WAXS beamline | | | | | | |
| detector | PILATUS 1M (Dectris) | | | | | | |
| wavelength (Å) | 1.0332 | | | | | | |
| Maximum flux at sample | 8 x 10^12^ photons per second at 12 keV | | | | | | |
| Camera length (mm) | 1600 | | | | | | |
| Q range (Å^-1^) | 0.006-0.5 | | | | | | |
| Exposure time | Continuous 1 second frame measurements | | | | | | |
| Sample configuration | SEC-SAXS with co-flow | | | | | | |
| Sample temperature (°C) | 12 | | | | | | |

| **Data collection statistics** | **DHDPS + lysine** | **DHDPS + pyruvate** | **DHDPS + pyruvate/SSA** |
| --- | --- | --- | --- |
| wavelength (Å) | 0.95369 | 0.95369 | 0.95369 |
| space group | C2 | C2 | C2 |
| unit cell parameters (a, b, c, Å) | 101.9, 133.5, 155.8 | 101.8, 133.6, 156.0 | 101.1, 132.9, 154.9 |
| resolution range (Å) | 45.2–2.01 (2.08–2.01) | 46.3–2.40 (2.49–2.40) | 45.8–1.93 (1.96–1.93) |
| observed reflections | 535,866 (53,764) | 317,539 (30,868) | 596,814 (26,666) |
| unique reflections | 135,620 (13,469) | 79,992 (7,961) | 151,510 (6,831) |
| mean *I/σ(I)* | 13.5 (1.2) | 8.0 (1.0) | 7.8 (0.9) |
| completeness (%) | 99.9 (99.5) | 99.8 (99.8) | 99.5 (91.1) |
| *R*_merge_ | 0.113 (0.553) | 0.344 (0.776) | 0.082 (1.25) |
| *R*_meas_ | 0.131 (0.644) | 0.394 (0.900) | 0.11 (1.63) |
| *R*_pim_ | 0.064 (0.315) | 0.191 (0.448) | 0.07 (1.11) |
| CC_½_ | 0.954 (0.802) | 0.920 (0.702) | 0.998 (0.468) |
| Wilson *B-*factor (Å^2^) | 31.4 | 36.9 | 36.7 |
| **Refinement statistics** |  |  |  |
| *R*_factor_ | 0.172 (0.239) | 0.199 (0.282) | 0.185 (0.400) |
| *R*_free_ | 0.209 (0.281) | 0.229 (0.301) | 0.225 (0.430) |
| number of atoms |  |  |  |
| non-hydrogen | 14,217 | 13,864 | 14,476 |
| macromolecules | 13,464 | 13,416 | 13,489 |
| solvent | 753 | 488 | 978 |
| protein residues | 1,776 | 1,776 | 1,776 |
| r.m.s.d. bonds (Å), angles (°) | 0.008, 1.0 | 0.022, 1.6 | 0.007, 1.0 |
| Ramachandran plot |  |  |  |
| favoured, outliers (%) | 98.4, 0.3 | 97.6, 0.1 | 97.6, 0.2 |
| rotamer outliers (%) | - | 1.2 | - |
| clash score | 2.53 | 4.08 | 3.62 |
| PDB id | 7LVL | 7LOY | 8GEK |

**Supplementary Table iv |** Data collection and refinement statistics for *C*LsoDHDPS with bound ligands. Values for the highest resolution shells are given in parentheses. Here, succinic semi-aldehyde is abbreviated to SSA.

**åSupplementary Table v** | Genes and primer sequences used in gene expression analysis.

| **Gene** | **Accession number** | **Protein** | **Primer sequence** |
| --- | --- | --- | --- |
| *dapA* | KJZ81861.1 | dihydrodipicolinate synthase | 5’-CACGGAGGTGTGGGTTGTAT-3’ 3’-GTGCTTGACGATAATCCCCCT-5’ |
| *recA* | KJZ80672 | recombinase A | 5’-TACGCCCTTTGGGAAAACCA-3’ 3’-TGCAGCTTGGCTCTCAAAGT-5’ |
| *Rpb* | KJZ81365.1 | DNA-directed RNA polymerase beta subunit | 5’-CCACAAGATACAATCGCCGC-3’ 3’-GCGGGAAAAGGTTTACTGCG-5’ |

**Supplementary Table vi** | Primers used to determine *Ca*. L. solanacearum titres in DNA samples and to serve as internal psyllid controls.

| **Primer** | **Primer sequence** |
| --- | --- |
| *C*LsoF | 5’-GTCGAGCGCTTATTTTTAATAGGA-3’ |
| *C*Lso16SF | 5’-ATACCGTATACGCCCTGAGAAG-3’ |
| *C*Lso16SRI | 3’-CGTAGCCTTGGTAGGCATT-5’ |
| ITS2F | 5’-AAGCGACGTGTGGAAGAACC-3’ |
| ITS2R | 3’-GTTGTGTGTGTCCGGGGAAG-5’ |
| COX-F | 5’-CGTCGCATTCCAGATTATCCA-5’ |
| COX-R | 3’-CAACTACGGATATATAAGAGCCAA AAC-5’ |

**Supplementary Table vii |** Comparison of first derivative (*T*_m_^D^) melting temperatures for *C*Lso pyruvate kinase (*C*LsoPykF) and *E. coli* pyruvate kinase (*Ec*PykF). Δ*T*_m_^D^ apo refers to the difference in *T*_m_^D^ when combinations of substrates and allosteric effector are added and compared to the apo *T*_m_^D^. The ligand concentrations were 5 mM. Errors represent standard errors.

|  | ***C*LsoPykF** (*T*_m_^D^, °C) | | | ***C*LsoPykF - *Ec*PykF** | ***Ec*PykF** (*T*_m_^D^, °C) | |
| --- | --- | --- | --- | --- | --- | --- |
| apo | 48.6 ± 0.2 | | | -11.8 | 60.4 ± 0.1 | |
| **+ ligand** |  | | **Δ*T*_m_^D^ apo** |  |  | **Δ*T*_m_^D^ apo** |
| ADP | 49.6 ± 0.1 | | 1.0 | -10.5 | 60.1 ± 0.1 | -0.3 |
| PEP | 45.7 ± 0.1 | | -2.9 | -14.4 | 60.1 ± 0 | -0.3 |
| FBP | 44.5 ± 0.3 | | -4.1 | -10.1 | 54.6 ± 0.1 | -5.8 |
| > 7.0 °C | |  |  |  |  |  |
| 7.0 – 4.0 °C | |  |  |  |  |  |
| 4.0 – 1.0 °C | |  |  |  |  |  |
| 1.0 – -1.0 °C | |  |  |  |  |  |
| -1.0 – -4.0 °C | |  |  |  |  |  |
| -4.0 – -7.0 °C | |  |  |  |  |  |
| > -7.0 | |  |  |  |  |  |

**Supplementary Figures**


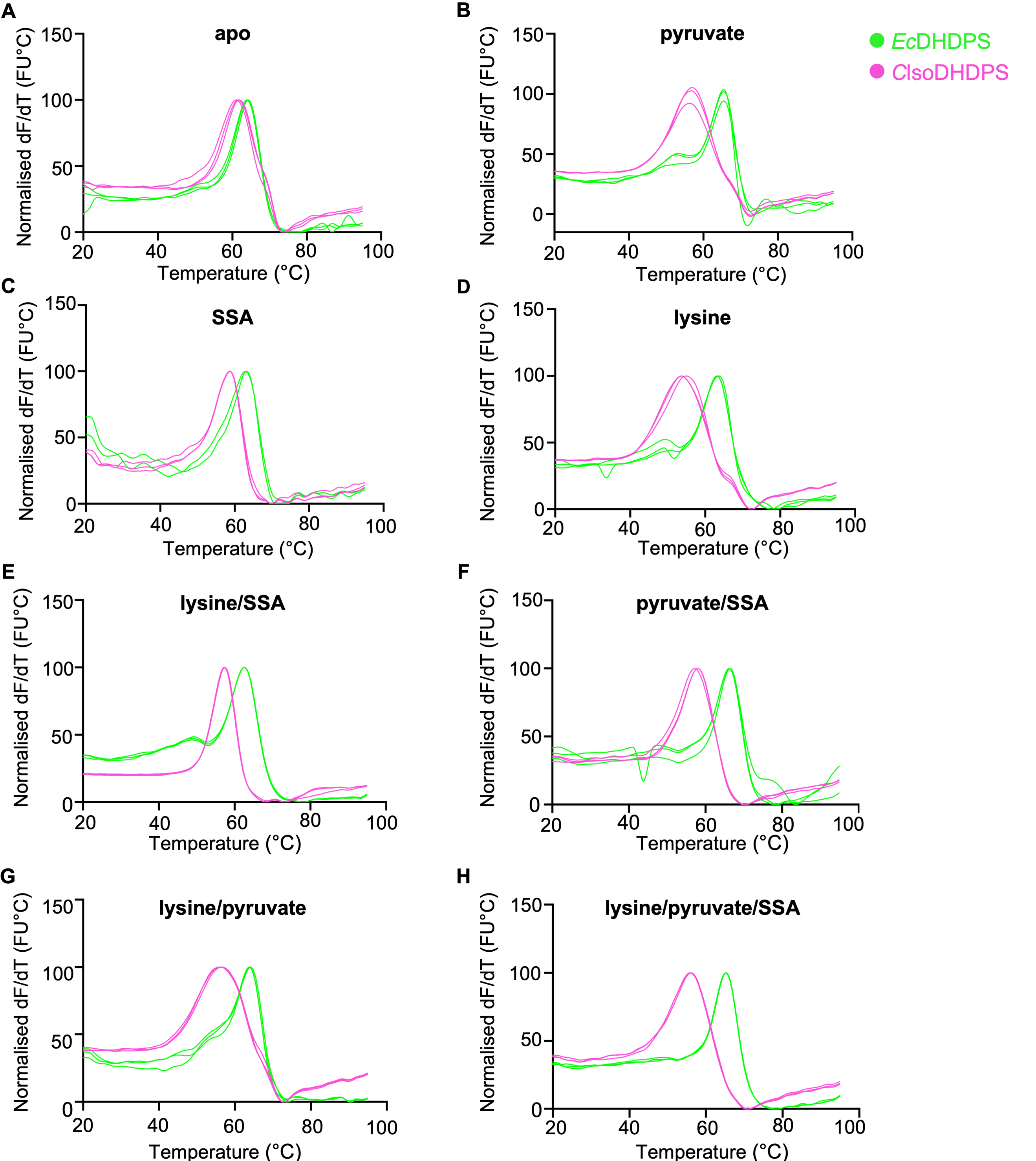
**Supplementary Figure i |** First derivative plots of differential fluorescence scanning data for *C*lsoDHDPS (pink) and *Ec*DHDPS (green).

**
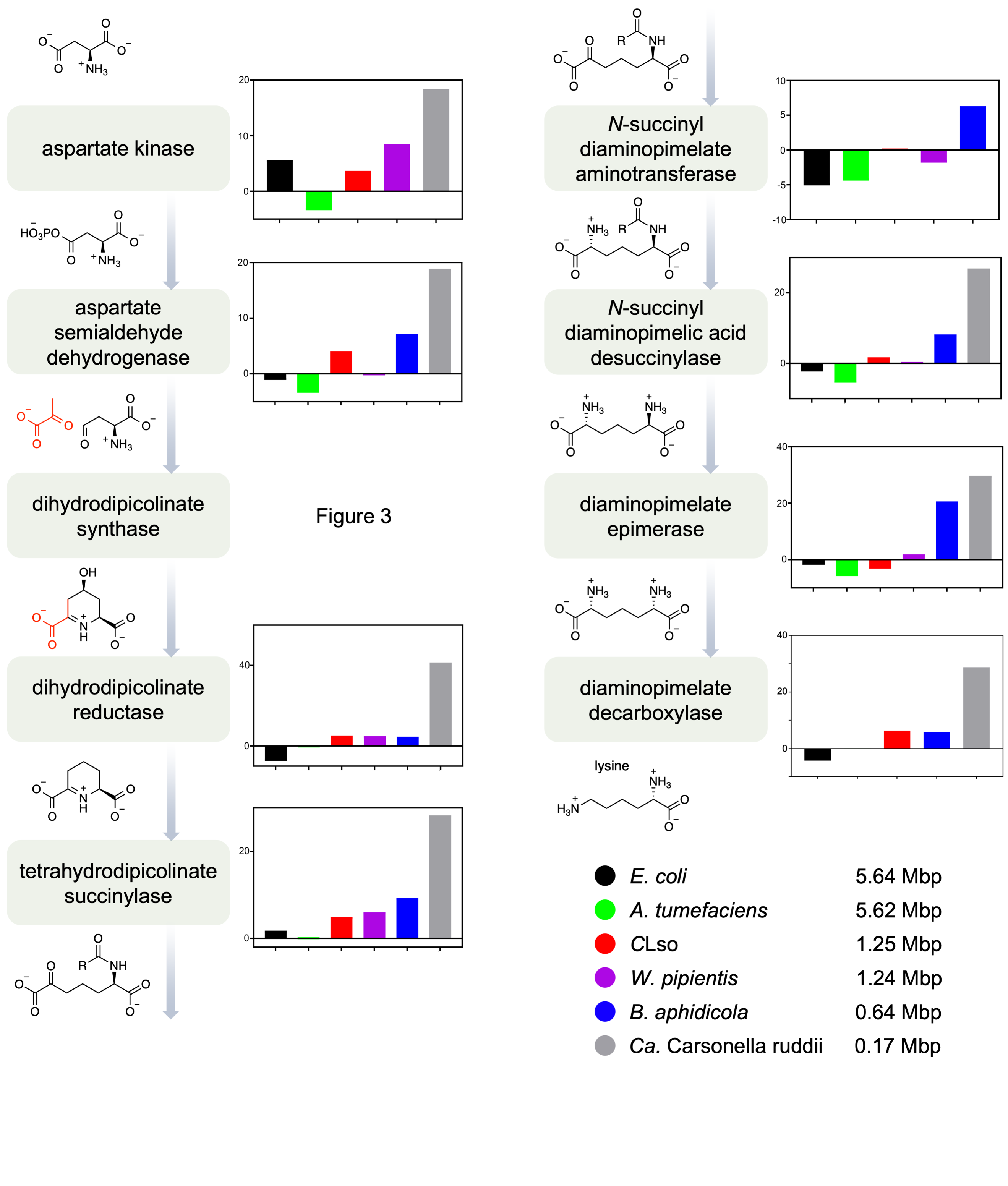
Supplementary Figure ii |** Aggregation propensity of enzymes in the lysine biosynthesis pathway highlight the trend of increased aggregation as the genome size is reduced. An increased positive Na^4^vSS score means an increase in the aggregation propensity for the protein, whereas a negative number suggests a decreased aggregation propensity.

**
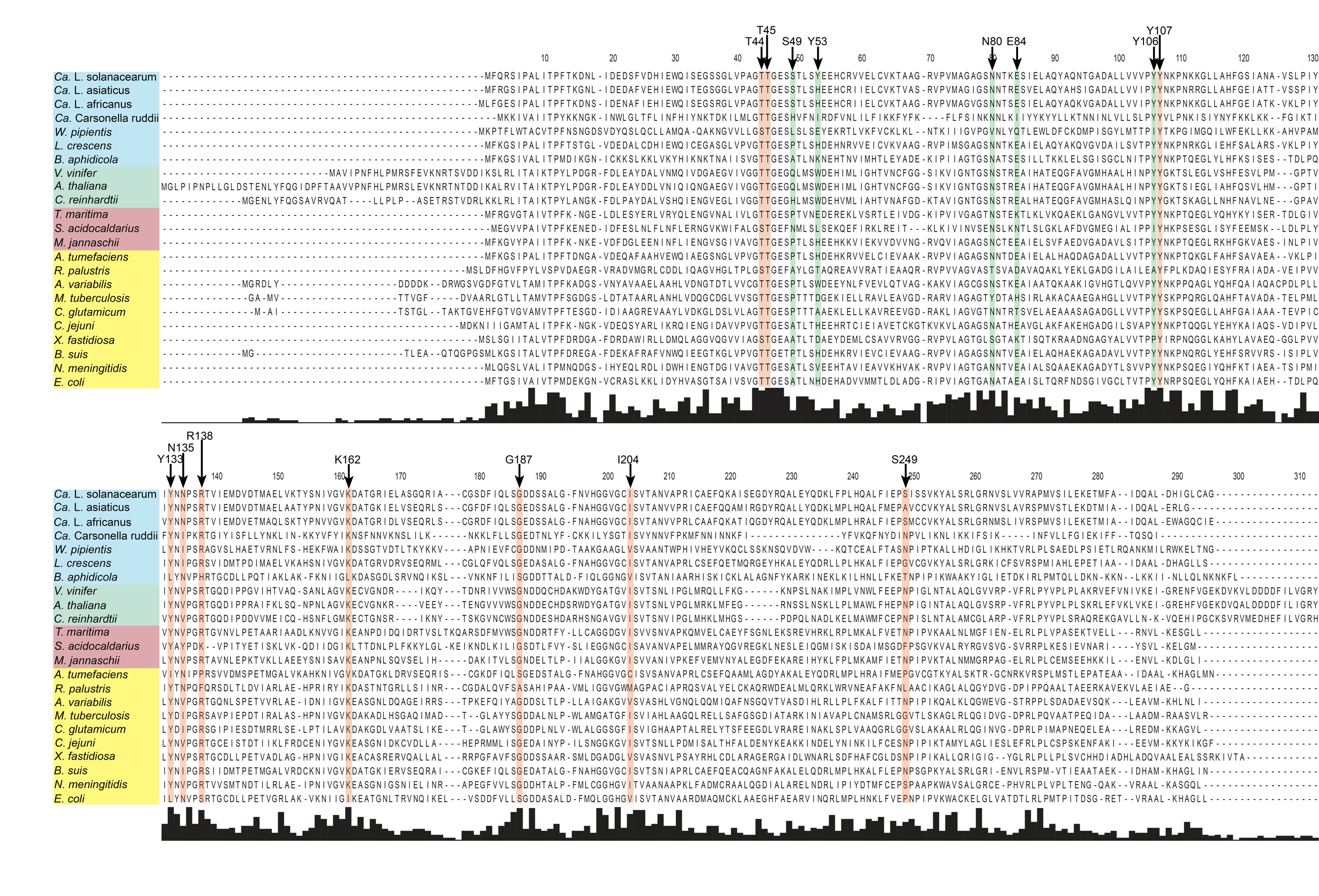
Supplementary Figure iii | Sequence alignments of *C*LsoDHDPS (top sequence) with representative bacterial and plant homologues.** Blue resents sequences from reduced genome bacteria, green from plant/algal, pink from extremophile bacteria, and yellow from mesophilic bacteria. Residues shaded in green represent those that fin lysine in the allosteric site, and those shaded on orange are active site residues that interaction with the substrates


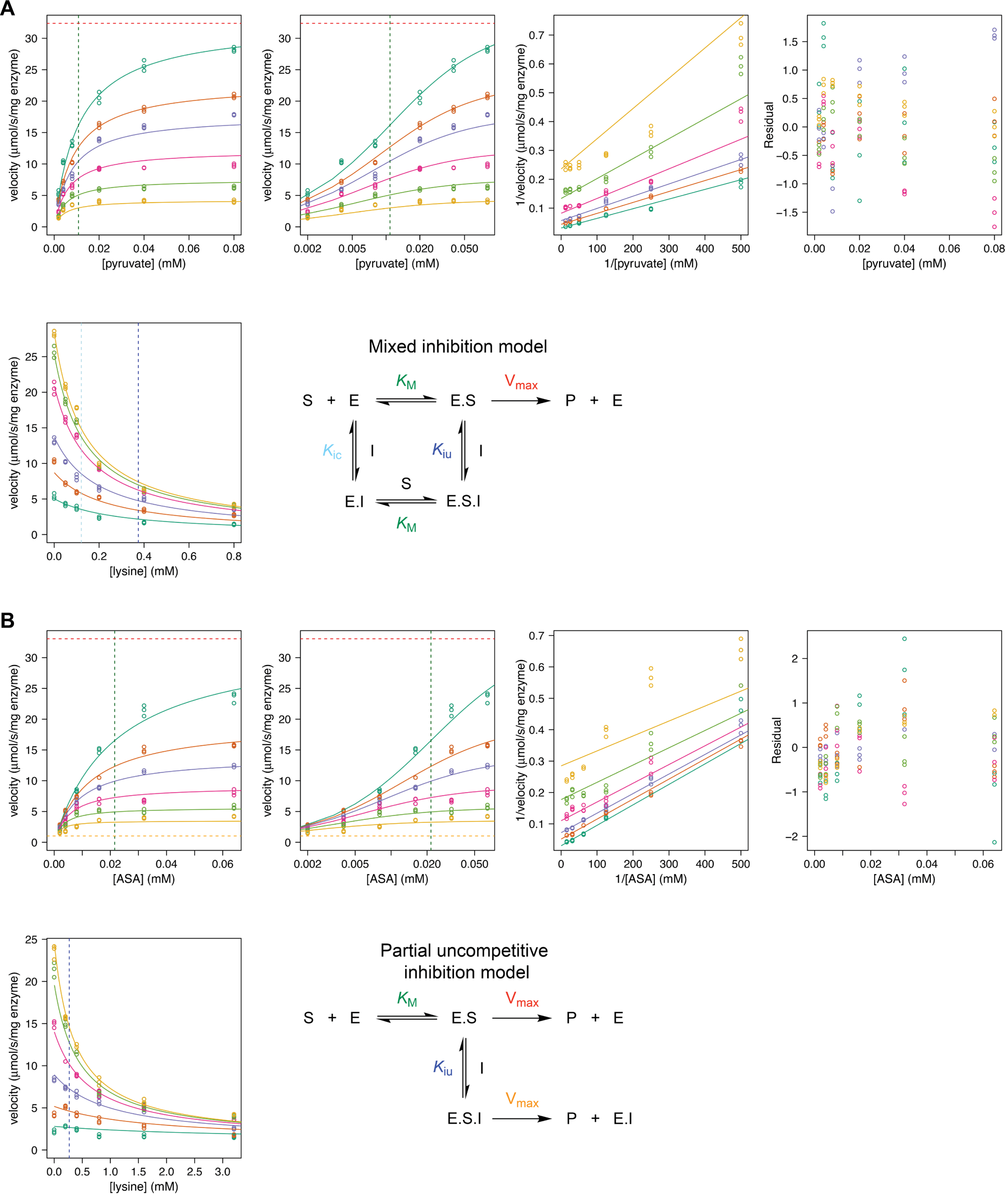
**Supplementary Figure iv | *C*LsoDHDPS is inhibited by lysine. A** is with respect to pyruvate at different lysine concentrations fitted to a mixed model inhibition. From left to right (first row) the plots include a direct plot, the direct plot with the substrate concentration on the log scale, the Lineweaver Burk plot, and the residuals of the fit. The second row displays a direct plot with respect to lysine at different concentrations of pyruvate as well as schematic representations of the mixed inhibition model. **B** is with respect to *S*-aspartate-β-semialdehyde (ASA) at different lysine concentrations fitted to a partial uncompetitive inhibition model. From left to right (first row) the plots include a direct plot, the direct plot with the substrate concentration on the log scale, the Lineweaver Burk plot, and the residuals of the fit. The second row displays a direct plot with respect to lysine at different concentrations of *S*-aspartate-β-semialdehyde (ASA) as well as a schematic representation of the partial uncompetitive inhibition models. In both A and B, the red dotted line shows the fitted *V*_max_^app^, the green dotted line is the *K*_M_^app^, and the blue dotted line is the *K*_i_^lysine^. All fits and plots were generated using R.

**
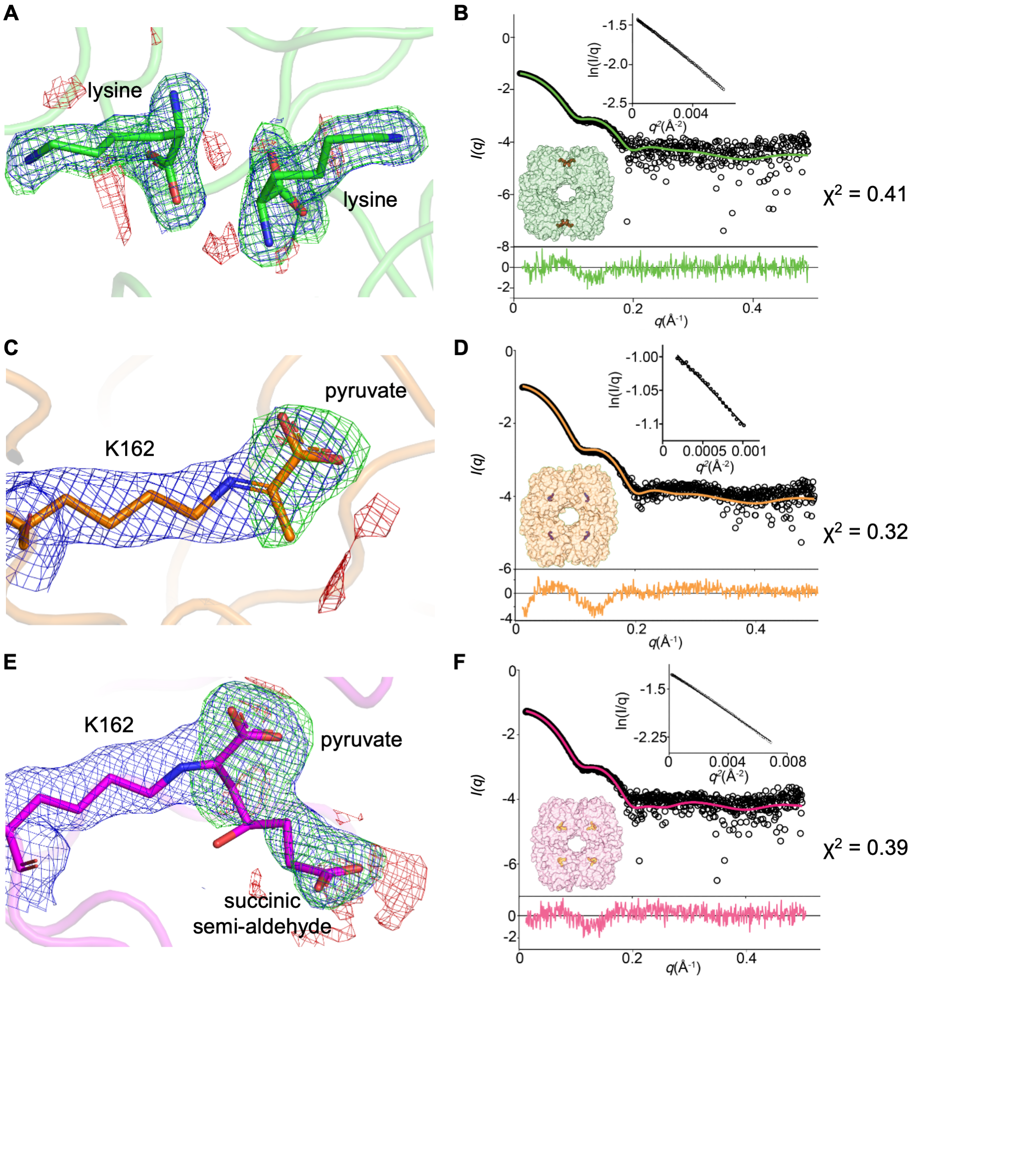
Supplementary Figure v | Omit maps and small angle X-ray scattering analysis of *C*LsoDHDPS with ligands.** **A, C** and **E.** Omit maps around the ligands bound to *C*LsoDHDPS [**A**, lysine (green); **C**, pyruvate (orange); and **E**, pyruvate + succinic semi-aldehyde (magenta)]. For all electron density maps, the 2Fo-Fc map is set to 1 σ (blue mesh), and the difference density Fo-Fc map is set to 3 σ (green mesh) and -3 σ (red mesh). **B, D** and **F.** Experimental scatter (ο) of *C*LsoDHDPS with ligands (**B**, lysine, 10 mM; **D**, pyruvate 5 mM; and **E**, pyruvate + succinic semi-aldehyde, 5 mM) fitted to the back calculated scatter from the equivalent ligand bound tetrameric crystal structures of *C*LsoDHDPS. The residuals for the fit are shown (bottom) along with the surface structure of *C*LsoDHDPS showing ligands (red sticks, bottom left) and the Guinier plot (inset top right). The χ^2^ value for each fit is indicated on the right.

**
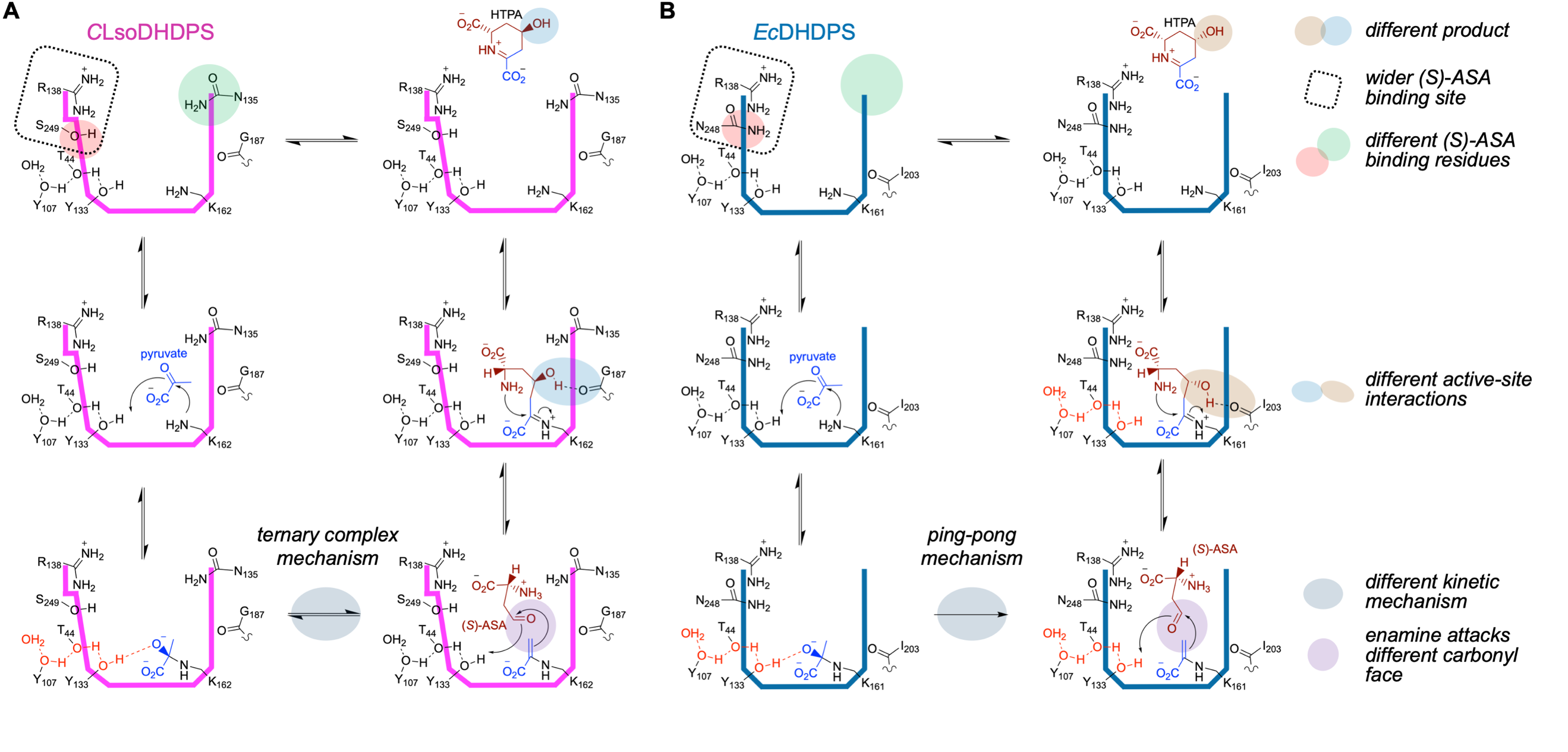
Supplementary Figure vi | Schematic highlighting the differences in the proposed mechanisms for *C*LsoDHDPS and *Ec*DHDPS.**

**Supplementary References**

Devenish SRA, Huisman FHA, Parker EJ, Hadfield AT, Gerrard JA. 2009. Cloning and characterisation of dihydrodipicolinate synthase from the pathogen Neisseria meningitidis. *Biochim Biophys Acta Proteins Proteom* 1794:1168–1174.

Dogovski C, Gorman MA, Ketaren NE, Praszkier J, Zammit LM, Mertens HD, Bryant G, Yang J, Griffin MDW, Pearce FG, et al. 2013. From knock-out phenotype to three-dimensional structure of a promising antibiotic target from Streptococcus pneumoniae. *PLoS One* 8:e83419.

Domigan LJ, Scally SW, Fogg MJ, Hutton CA, Perugini MA, Dobson RCJ, Muscroft-Taylor AC, Gerrard JA, Devenish SRA. 2009. Characterisation of dihydrodipicolinate synthase (DHDPS) from Bacillus anthracis. *Biochim Biophys Acta Proteins Proteom* 1794:1510–1516.

Griffin MDW, Billakanti JM, Wason A, Keller S, Mertens HDT, Atkinson SC, Dobson RCJ, Perugini MA, Gerrard JA, Pearce FG. 2012. Characterisation of the first enzymes committed to lysine biosynthesis in Arabidopsis thaliana. *PLoS One* 7:e40318.

Gupta R, Hogan CJ, Perugini MA, Costa TPS da. 2018. Characterization of recombinant dihydrodipicolinate synthase from the bread wheat Triticum aestivum. *Planta* 248:381–391.

Halling SM, Stahly DP. 1976. Dihydrodipicolinic acid synthase of Bacillus licheniformis. Quaternary structure, kinetics, and stability in the presence of sodium chloride and substrates. *Biochim. Biophys. Acta (BBA) - Enzym.* 452:580–596.

Impey RE, Panjikar S, Hall CJ, Bock LJ, Sutton JM, Perugini MA, Costa TPS da. 2020. Identification of two dihydrodipicolinate synthase isoforms from Pseudomonas aeruginosa that differ in allosteric regulation. *FEBS J* 287:386–400.

Karsten WE. 1997. Dihydrodipicolinate synthase from Escherichia coli: pH dependent changes in the kinetic mechanism and kinetic mechanism of allosteric inhibition by L-lysine. *Biochemistry* 36:1730–1739.

Kefala G, Evans GL, Griffin MDW, Devenish SRA, Pearce FG, Perugini MA, Gerrard JA, Weiss MS, Dobson RCJ. 2008. Crystal structure and kinetic study of dihydrodipicolinate synthase from Mycobacterium tuberculosis. *Biochem J* 411:351–10.

Pearce FG, Perugini MA, Mckerchar HJ, Gerrard JA. 2006. Dihydrodipicolinate synthase from Thermotoga maritima. *Biochem J* 400:359–366.

Skovpen YV, Palmer DRJ. 2013. Dihydrodipicolinate synthase from Campylobacter jejuni: kinetic mechanism of cooperative allosteric inhibition and inhibitor-induced substrate cooperativity. *Biochemistry* 52:5454–5462.
